# Systems Drug Design for Muscle Invasive Bladder Cancer and Advanced Bladder Cancer by Genome-Wide Microarray Data and Deep Learning Method with Drug Design Specifications

**DOI:** 10.3390/ijms232213869

**Published:** 2022-11-10

**Authors:** Po-Wei Su, Bor-Sen Chen

**Affiliations:** Laboratory of Automatic Control, Signal Processing and Systems Biology, Department of Electrical Engineering, National Tsing Hua University, Hsinchu 30013, Taiwan

**Keywords:** muscle-invasive bladder cancer (MIBC), advanced bladder cancer (ABC), deep neural network (DNN)-based drug-target interaction (DTI) model, drug targets, drug design specifications, drug combination

## Abstract

Bladder cancer is the 10th most common cancer worldwide. Due to the lack of understanding of the oncogenic mechanisms between muscle-invasive bladder cancer (MIBC) and advanced bladder cancer (ABC) and the limitations of current treatments, novel therapeutic approaches are urgently needed. In this study, we utilized the systems biology method via genome-wide microarray data to explore the oncogenic mechanisms of MIBC and ABC to identify their respective drug targets for systems drug discovery. First, we constructed the candidate genome-wide genetic and epigenetic networks (GWGEN) through big data mining. Second, we applied the system identification and system order detection method to delete false positives in candidate GWGENs to obtain the real GWGENs of MIBC and ABC from their genome-wide microarray data. Third, we extracted the core GWGENs from the real GWGENs by selecting the significant proteins, genes and epigenetics via the principal network projection (PNP) method. Finally, we obtained the core signaling pathways from the corresponding core GWGEN through the annotations of the Kyoto Encyclopedia of Genes and Genomes (KEGG) pathway to investigate the carcinogenic mechanisms of MIBC and ABC. Based on the carcinogenic mechanisms, we selected the significant drug targets NFKB1, LEF1 and MYC for MIBC, and LEF1, MYC, NOTCH1 and FOXO1 for ABC. To design molecular drug combinations for MIBC and ABC, we employed a deep neural network (DNN)-based drug-target interaction (DTI) model with drug specifications. The DNN-based DTI model was trained by drug-target interaction databases to predict the candidate drugs for MIBC and ABC, respectively. Subsequently, the drug design specifications based on regulation ability, sensitivity and toxicity were employed as filter criteria for screening the potential drug combinations of Embelin and Obatoclax for MIBC, and Obatoclax, Entinostat and Imiquimod for ABC from their candidate drugs. In conclusion, we not only investigated the oncogenic mechanisms of MIBC and ABC, but also provided promising therapeutic options for MIBC and ABC, respectively.

## 1. Introduction

Bladder cancer is the 10th most common cancer worldwide, with 573,278 new cases and 212,536 deaths in 2020 [1]. Most bladder cancers are urothelial carcinomas. Bladder cancer is diagnosed as a muscle-invasive bladder cancer (MIBC) or an advanced bladder cancer (ABC) once the tumor has invaded the muscle layer of the bladder. At present, approximately 70% of diagnosed patients have non-muscle-invasive bladder cancer (NMIBC), and 30% have MIBC or ABC. Moreover, approximately 15% to 20% of NMIBCs will progress to MIBC, and half of MIBCs will undergo metastasis and die within 3 years [2,3].

Although there has been significant progress in other cancer treatments, the systemic treatment of bladder cancer has remained largely unchanged for more than 30 years. However, a better understanding of the oncogenic mechanisms and clinical drug discovery for bladder cancer have facilitated the rapid expansion of therapies in the past decade. Most bladder cancer patients are diagnosed after the appearance of macroscopic hematuria and cases are identified during the transurethral resection of bladder tumors (TURBTs) [4]. Currently, the standard treatment of MIBC is multimodal therapy, including radical cystectomy, neoadjuvant chemotherapy (NAC) or bladder-sparing trimodality therapy for the selected patients with MIBC, which contains transurethral resection and chemoradiotherapy; ABC is treated with cisplatin-based systemic chemotherapy [5]. In invasive bladder cancer, the major prognostic factor is staged based on the depth of the tumor invasion and metastasis [6]. The 5-year overall survival (OS) rate for patients with MIBC is roughly 60–70%. Even though NAC therapy has a 5–10% survival benefit, a certain percentage of patients have no response to NAC and may experience potentially fatal surgical delays and side effects from the treatment [7]. Conventional cisplatin-based chemotherapy has an initial high response rate for ABC, but the 5-year OS rate is only 5–20% [8]. Furthermore, chemoradiotherapy has failed to generally improve the prognosis of ABC because of patient resistance to the treatment [9]. Therefore, we need to look for better treatment options to prolong the opportunity for survival. The targeted therapy of bladder cancer malignancies, a revolutionary treatment that prevents cancer growth and metastasis by interfering with specific molecules, is actively being developed [10].

With the rapid development of cancer genomics, there are other treatments to eradicate bladder cancer cells that target abnormally expressed molecules in bladder cancer tissues. Long non-coding RNAs (lncRNAs) are defined as transcripts longer than 200 nucleotides [11]. They play key roles in post-transcriptional regulation mechanisms targeting mRNA splicing, stability or translation [12]. A recent study has demonstrated that the abnormally expressed lncRNAs can serve as oncogenes and tumor suppressors, which are related to tumor recurrence, metastasis, prognosis or diagnosis [13]. In addition, lncRNAs are involved in carcinogenic mechanisms of the genitourinary system, including prostate, renal cell and bladder malignancies. Therefore, lncRNAs are promising therapeutic candidate targets for molecular research and biomarker discovery in genitourinary oncology [14].

Another potential target of non-coding RNAs, microRNAs (miRNAs), are evolutionarily conserved and small non-coding RNAs that are approximately 22 nucleotides in length [15]. miRNAs regulate the expression of genes involved in the control of differentiation, apoptosis and proliferation [16]. The mechanism of miRNAs in bladder cancer is an emerging field of research. The study has shown that miRNA genes are dysregulated in cancer, and the alteration in miRNA expression can affect the cell cycle and survival [17]. miRNAs are potential prognostic and diagnostic biomarkers and may also serve as novel drug targets for the treatment of bladder cancer [18].

In traditional drug discovery, the development of a new drug is very expensive and time-consuming, taking approximately 13–15 years and costing an average of 2–3 billion USD from the lab to the market [19]. The approval rates for cancer drugs entering phase I trials are only 5% [20]. Therefore, we urgently need a more efficient and systematic drug design. The drug-target interaction (DTI) prediction can narrow down the scope of candidate drugs, hence, it has become an important process in drug discovery and drug repurposing [21]. The traditional computational methods based on the DTI model can be broadly classified into ligand-based approaches and docking-based approaches, which are used to predict the interactions based on the similarities between proteins and ligands [22]. With the development of computational applications and artificial intelligence, drug screening and design employ these methods to reduce the time and cost of drug discovery. In the past decade, machine learning-based DTI prediction methods have been rapidly developed. DTI prediction is generally considered a binary classification problem by utilizing the features of drugs and targets as inputs and the proved DTI as labels. Compared with traditional computational methods, network-based methods show great advantages from the concept that most drugs and compounds interact with multiple targets (biomarkers) [23]. Drug repurposing refers to the application of the drug for other indications than its initial approval. The advantage of drug repurposing is that large amounts of data can be obtained for investigation, and it can also reduce the need for additional studies on pharmacokinetic properties and toxicity [24]. Moreover, multiple drug combinations can target more biomarkers of the tumor as drug targets and have clinically improved tumor growth and survival in patients on monotherapy. Drug combinations can restore more cellular dysfunctions in tumors, so the efficacy of each drug in the combination can be enhanced without increasing the dose of a single drug. Since drug combination as a multiple-molecule drug can increase the efficacy of each molecular drug in the combination, it can achieve the same efficacy requirements with lower doses than monotherapy, thereby reducing toxicity, drug resistance and side effects in patients [25]. Therefore, we applied DTI prediction based on the deep neural network (DNN) for systems drug design and discovery and selected multiple-molecule drugs for the treatment of MIBC and ABC from the perspective of drug repurposing and drug combination.

In this study, we utilized the systems biology method via genome-wide microarray data for exploring the oncogenic mechanisms to identify drug targets and employed the DNN-based DTI model to predict candidate molecular drugs and then, with drug specifications, to select potential multiple-molecule drugs as drug combinations for the treatment of MIBC and ABC, respectively. First, we constructed the candidate genome-wide genetic and epigenetic network (GWGEN) with big data mining. Second, we applied the system identification scheme and system order detection approach to delete false-positive interactions or regulations in candidate GWGENs through the corresponding genome-wide microarray data to obtain the real GWGENs. Third, we extracted the core GWGENs from the real GWGENs by selecting the significant proteins, genes and epigenetics via the principal network projection (PNP) method. We could then, obtain the core signaling pathways from the corresponding core GWGEN through the annotations of the Kyoto Encyclopedia of Genes and Genomes (KEGG) pathway to investigate the significant carcinogenic mechanisms leading to abnormal cellular functions of MIBC and ABC. Based on the corresponding carcinogenic mechanisms, we selected the significant biomarkers NFKB1, LEF1 and MYC as drug targets for MIBC, and the significant biomarkers LEF1, MYC, NOTCH1 and FOXO1 as drug targets for ABC. Finally, the DNN-based DTI model was trained with drug-target interaction databases to predict the candidate molecular drugs for the drug targets of MIBC and ABC. Subsequently, the drug design specifications based on regulation ability, sensitivity and toxicity were employed as filter criteria for screening the potential drug combination of Embelin and Obatoclax as the multiple-molecule drug for MIBC, and the potential drug combination of Obatoclax, Entinostat and Imiquimod as the multiple-molecule drug for ABC from their candidate molecular drugs to provide promising therapeutic options, respectively.

## 2. Results

### 2.1. Overview of Systems Biology Approach for the Investigation of Carcinogenic Mechanism and Systematic Drug Design for the Treatment of MIBC and ABC

**Figure 1:**
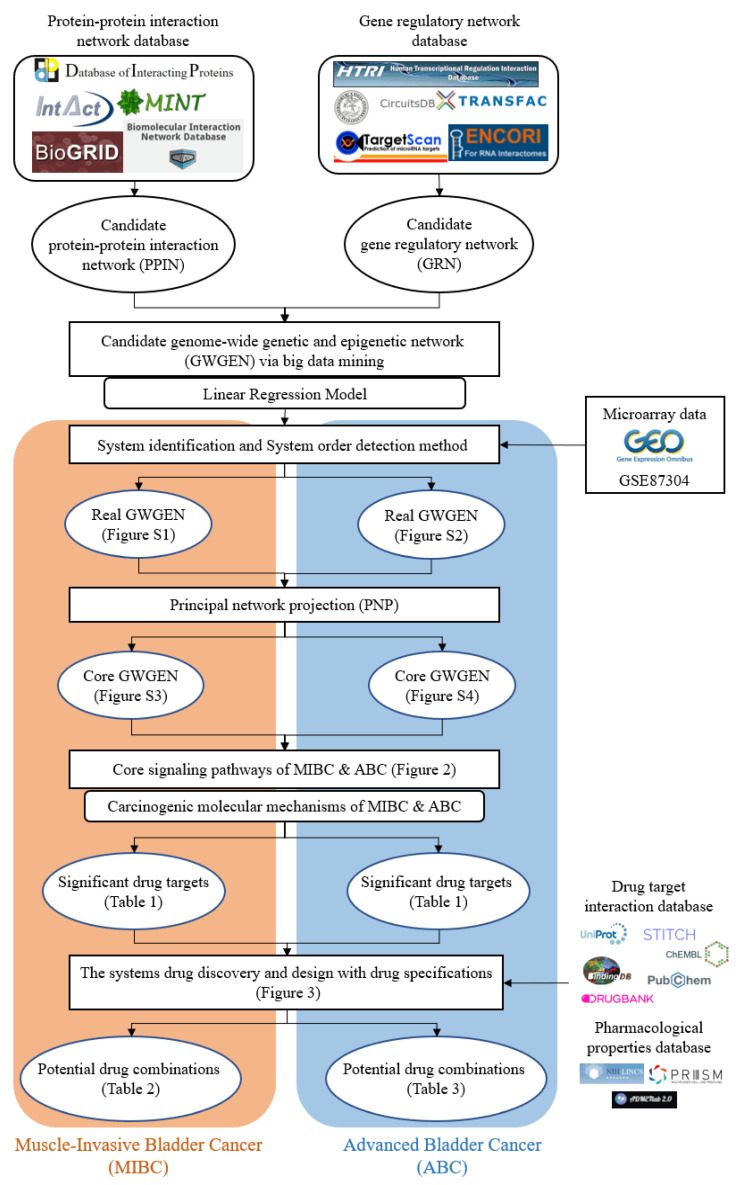
The flowchart of the systems biology method and systematic drug discovery design. The construction of candidate GWGEN, real GWGEN, core GWGEN and core signaling pathways for investigating carcinogenic mechanisms to identify the biomarkers as drug targets of MIBC and ABC, and systematic drug discovery and design of potential drug combinations as multiple-molecule drugs to target the corresponding multiple drug targets for the treatment of MIBC and ABC.

**Figure 2:**
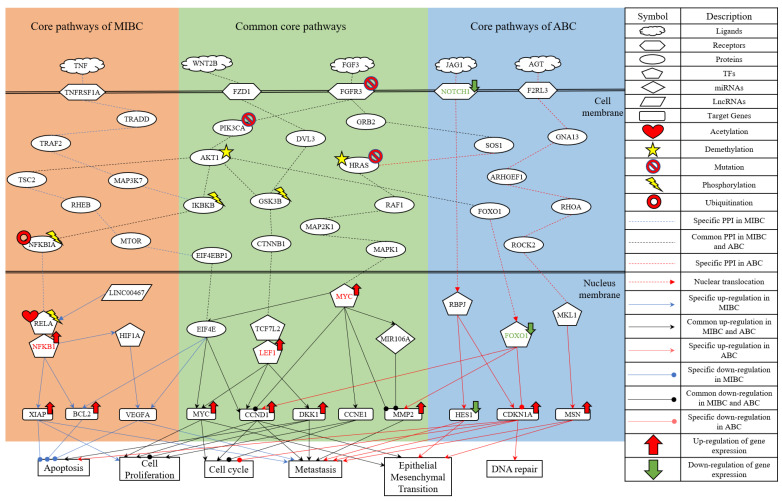
The common and specific core signaling pathways and their downstream cellular dysfunctions between MIBC and ABC. The figure shows the genetic and epigenetic carcinogenic mechanisms of MIBC and ABC. The orange background contains the specific core signaling pathways of MIBC. The green background contains the overlapping core signaling pathways between MIBC and ABC (i.e., common core signaling pathways). The blue background contains the specific core signaling pathways of ABC. The gene symbols in red or green font denote the selected significant biomarkers as drug targets.

In this research, we utilized the systems biology method to investigate the molecular carcinogenesis mechanism with genome-wide microarray data and applied the DNN-based DTI model with drug design specifications for systematic drug discovery to predict potential drug combinations as multiple-molecule drugs for the treatment of MIBC and ABC, respectively. The flowchart of the systems biology approach to identify the biomarkers of carcinogenic mechanisms as drug targets for drug discovery of MIBC and ABC is shown in Figure 1. After constructing the candidate GWGEN via big data mining, based on a protein interaction and gene regulation model, we applied the corresponding genome-wide microarray data by the system identification and the system order detection methods to identify the real GWGENs of MIBC and ABC, respectively. The nodes of the candidate and real GWGENs are divided into five groups: proteins, receptors, transcription factors (TFs), miRNAs and lncRNAs. The statistics of nodes and edges in the candidate GWGENs and the real GWGENs after system identification are individually given in Tables S1 and S2. It is noted that, compared with candidate GWGENs, the total number of edges in the real GWGENs is significantly reduced. This result indicates that the false-positive interactions or regulations from datasets under different experimental conditions and noises can be pruned by the system order detection approach. Although the scale of the GWGEN networks of MIBC and ABC has shrunk, the real GWGENs of MIBC and ABC in Figures S1 and S2 are still too complex to be precisely analyzed and annotated by KEGG pathways for the significant carcinogenic mechanisms of MIBC and ABC, respectively. Hence, we performed the PNP method on the real GWGENs of MIBC and ABC to separately extract their core GWGENs based on the projection values of the nodes in their real GWGENs. In this study, the core GWGENs contain the top-ranked 6000 nodes that reflect significant networks of the real GWGENs from an 85% network energy perspective. We plotted the real GWGENs and core GWGENs of MIBC and ABC with the network visualization software Cytoscape in Figures S1–S4, respectively. Moreover, the KEGG pathway annotations for the core GWGENs of MIBC and ABC are obtained based on the core nodes through the enrichment analysis of the database for annotation, visualization and integrated discovery (DAVID) to explore the genetic and epigenetic carcinogenic mechanisms of MIBC and ABC. The enrichment analysis results of MIBC and ABC are shown in Tables S3 and S4, respectively. According to KEGG signaling pathway annotations and related literature surveys, we established the common and specific core signaling pathways for MIBC and ABC, and discussed the involved carcinogenic mechanisms, as shown in Figure 2. The significant biomarkers of MIBC and ABC were then identified as drug targets by investigating the core signaling pathways leading to abnormal cellular functions of MIBC and ABC, respectively. For drug discovery and design, we constructed a DNN-based DTI model to predict the candidate drugs for the drug targets of MIBC and ABC. On the basis of drug design specifications, such as regulation ability, sensitivity and toxicity, the candidate drugs were further screened for potential drug combinations as multiple-molecule drugs of MIBC and ABC. The following sections provide more details on the core signaling pathways and the systematic drug discovery design.

### 2.2. The Specific Molecular Carcinogenic Mechanisms of MIBC

Based on the projection values of each node in the core GWGEN of MIBC, we selected and investigated the specific signaling pathways of MIBC with the blue lines in Figure 2. The receptor TNFRSF1A receives the microenvironment factor TNF to activate TF NFKB1/RELA through signaling transduction proteins TRADD, TRAF2, MAP3K7, IKBKB and NFKBIA. The protein IKBKB is phosphorylated upon stimulation, which in turn phosphorylates the protein NFKBIA and causes its ubiquitination and degradation. TF NFKB1 is then released into the nucleus to initiate gene transcription. Furthermore, protein AKT is an upstream regulator of protein IKBKB and utilizes IKBKB to activate NF-κB transactivation, which initiates RELA phosphorylation [26]. LncRNA LINC00467 is the upstream node of TF RELA. It has been confirmed that LINC00467 can directly bind to TF RELA and activate the NF-κB signaling pathway to promote the development and occurrence of bladder cancer [27]. TF RELA can be acetylated to increase the DNA binding of NF-κB [28]. TF NFKB1 can positively regulate the target genes *XIAP* and *BCL2,* while inducing the activation of TF HIF1A [29,30,31]. Overexpression of *XIAP* and *BCL2* inhibits the apoptosis of cancer cells, and *XIAP* also enhances human invasive bladder cancer cell proliferation [32,33,34]. Mediated by TF NFKB1, TF HIF1A can induce the target gene *VEGFA* [35]. *VEGFA* is a factor of endothelial cells during tumor angiogenesis and functions in antiapoptosis and vasodilation [36]. *VEGFA* is associated with prognosis in patients with MIBC and is also thought to increase the metastasis of cancer cells [37].

In summary, the specific molecular carcinogenic mechanisms of MIBC are due to abnormal cellular functions of apoptosis, cell proliferation and metastasis, which promote the progression of MIBC. According to the analysis of core signaling pathways for MIBC and considering the protein/gene expression levels [38], the significant biomarker TF NFKB1 is selected as the drug target for the specific oncogenic mechanism of MIBC.

### 2.3. The Common Molecular Mechanisms between MIBC and ABC

The common core signaling pathways between MIBC and ABC are displayed with black lines in Figure 2. After binding to the ligand FGF3, the receptor FGFR3 can separately trigger the signaling transduction proteins PIK3CA and GRB2. The protein PIK3CA can activate the translation initiation factor EIF4E through the mediation of the PI3K/AKT/mTOR signaling pathway. The previous study reported mutations in the receptor FGFR3 and protein PIK3CA in bladder cancer [39]. Additionally, the AKT1 promoter is nearly demethylated in bladder cancer tissues, and the methylation level of AKT1 is found to be clinically correlated with bladder cancer [40]. Subsequently, EIF4E can induce the target genes *BCL2*, *VEGFA*, *MYC* and Cyclin D1 (*CCND1*) [41,42]. Overexpression of *MYC* and *CCND1* is involved in many cellular functions, such as cell cycle enhancement, cell proliferation initiation and epithelial-mesenchymal transition (EMT) promotion [43,44,45,46,47,48]. Furthermore, the study has revealed that the overexpression of *CCND1* is an independent factor in the metastasis of bladder cancer [49].

After the receptor FZD1 interacts with the ligand WNT2B, it can activate the Wnt/β-catenin signaling pathway, as shown in Figure 2. The Wnt/β-catenin signaling pathway utilizes the protein CTNNB1 to transmit the receptor-mediated signaling from the cytoplasm to the nucleus, where it activates the TFs TCF/LEF [50]. TFs TCF/LEF can induce the transcription of target genes *MYC*, *CCND1* and *DKK1* [51,52]. The overexpression of *Dkk1* can facilitate apoptosis by suppressing cell proliferation and transformation, which occurs in multiple cancer cell lines, such as lung cancer, cervical cancer and liver cancer [53]. Moreover, the high-level expression of *Dkk1* in preoperative advanced bladder cancer patients is closely associated with tumor stage, poor patient survival and distant metastasis [53,54].

The previously described receptor FGFR3 triggers not only the PI3K/AKT/mTOR signaling pathway, but also the MAPK signaling pathway. Activation of MAPK1 is a common event in tumor metastasis. Mutations in HRAS are found in bladder cancer and can serve as a tumor staging function. Expression of HRAS with activating mutations in urothelial cells results in superficial tumors and urothelial hyperplasia [55]. Moreover, the aberrant activation of HRAS during bladder cancer development may be caused by the demethylation of the HRAS promoter [56]. TF MYC can separately inhibit target gene *CCND1*, induce target gene *CCNE1*, activate EIF4E and trigger miRNA MIR106A through the mediation of the MAPK signaling pathway [57,58,59,60]. Transcriptional control of *CCNE1* regulates the cell cycle in bladder cancer, so its aberrant expression leads to rapid proliferation and genomic instability [61]. Transfection of miRNA MIR106A inhibits the target gene *MMP2*. *MMP*s play an important role in cancer cell invasion by degrading basement membranes and the cellular matrix [62]. Upregulation of *MMP2* is a significant process in the invasion and migration of bladder cancer cells [63].

In brief, the common molecular carcinogenic mechanisms between MIBC and ABC are due to abnormal cellular functions of apoptosis, cell proliferation, cell cycle, metastasis and EMT. Based on the results of the core signaling pathways and the discussion of protein/gene expressions [64,65], the significant biomarkers TF LEF1 and TF MYC are chosen as the drug targets for the common oncogenic mechanisms between MIBC and ABC.

### 2.4. The Specific Molecular Carcinogenic Mechanisms of ABC

The specific core signaling pathways of ABC are shown as red lines in Figure 2**.** NOTCH1 is a single-pass transmembrane receptor that is triggered by interacting with ligands in JAG1 on an adjacent cell membrane. The NOTCH intracellular domain translocates to the nucleus, where it activates TF RBPJ [66]. TF RBPJ induces the transcription of target genes *HES1* and *CDKN1A* [67,68]. The low-expression level of *HES1* is associated with EMT and metastatic properties in bladder cancer [66]. The overexpression of *CDKN1A* can promote apoptosis, cell cycle arrest and bladder cancer metastatic ability [69,70,71]. In addition, *CDKN1A* can reduce drug sensitivity by enhancing DNA repair [72].

The previously described protein AKT1 can also negatively regulate the protein FOXO1 after the receptor FGFR3 interacts with the ligand FGF3. The abnormal expression of FOXO1 was observed in metastatic bladder cancer cells and it was associated with poorer outcomes and recurrence in bladder cancer [73]. The down-regulated expression of TF FOXO1 can induce target genes *CCND1* and *MMP2* but inhibit target gene *CDKN1A* [74,75,76].

After the ligand AGT binds to the receptor F2RL3, it initiates the Rho/ROCK pathway as shown in Figure 2. The Rho/ROCK pathway is involved in bladder cancer invasion, metastasis and progression by regulating actin cytoskeleton organization and it may be a valuable prognostic biomarker [77]. TF MKL1, activated by the upstream protein ROCK2, can regulate the target gene *MSN* [78]. *MSN* as a potential biomarker for predicting advanced bladder cancer has been found to have higher expression associated with poor survival in cancer. Accumulating evidence indicates that *MSN* plays a key role in the EMT of various malignant tumors [79].

In conclusion, the specific molecular carcinogenic mechanisms of ABC are due to abnormal cellular functions of apoptosis, cell cycle, metastasis, EMT and DNA repair. Based on the analysis of the core signaling pathways for ABC and considering the protein/gene expressions [75,80], the biomarkers NOTCH1 and TF FOXO1 are selected as the drug targets for the specific oncogenic mechanisms of ABC.

### 2.5. The Deep Neural Network-Based Drug-Target Interaction Model with Drug Design Specifications to Discover the Potential Drug Combinations for Multiple-Molecule Drugs of MIBC and ABC

Based on the investigation of oncogenic mechanisms of MIBC and ABC in the previous sections, we separately chose biomarkers NFKB1, LEF1 and MYC, and biomarkers LEF1, MYC, NOTCH1 and FOXO1 as drug targets for MIBC and ABC, and then searched for small compounds that could be suitable for potential drug combination therapies with adequate regulation ability, sensitivity and toxicity to reverse the expression levels of the biomarkers.

**Figure 3:**
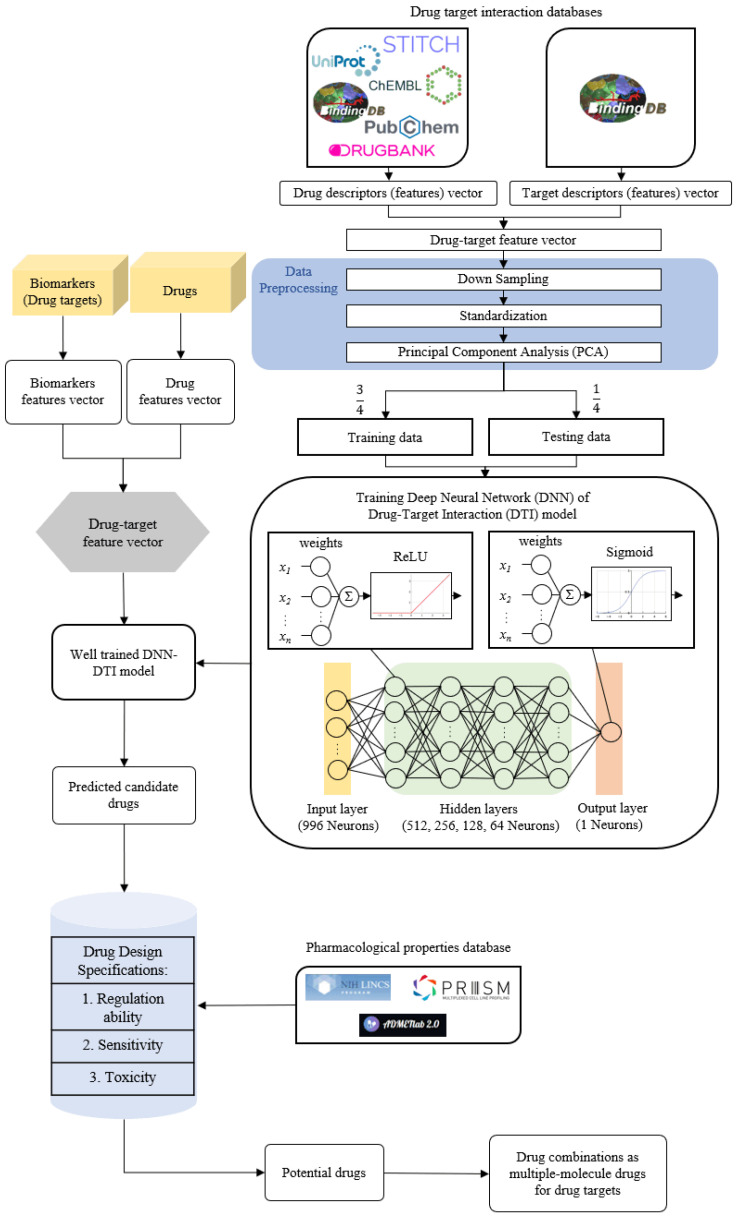
The flowchart of systematic drug design and discovery of MIBC and ABC. The drug-target interaction databases contain drug-target interaction data to construct the drug-target feature vector. After data preprocessing, the data is divided into training data and testing data to train the DNN-based DTI model. The feature vectors of biomarkers and drugs from drug-target interaction databases are used for the well-trained DNN-based DTI model to predict candidate drugs for the identified biomarkers (drug targets) of MIBC and ABC. The candidate drugs are then filtered by the drug design specifications to obtain potential drug combinations as multiple-molecule drugs for the treatment of MIBC and ABC.

In order to construct the systematic drug design and discovery process, we pretrained a DNN-based DTI model to efficiently predict the interaction probability between candidate drugs and the identified drug targets. The potential drug combinations as multiple-molecule drugs for MIBC and ABC were then filtered from the candidate drugs by the drug design specifications of regulation ability, sensitivity and toxicity. The flowchart of systematic drug design and discovery is shown in Figure 3. There are a total of 80,291 proven drug-target interactions and 100,024 unproven drug-target interactions in our training datasets. To avoid poor prediction problems caused by an imbalanced class distribution, the model is overwhelmed by the large class and ignores the minority class. We randomly selected an equal number of proven and unproven drug-target interactions. Since the features in drug-target interactions are defined in different units and the datasets in high-dimensional space are complex for DNN-based DTI model training, we performed feature scaling by the standardization method and utilized the principal component analysis (PCA) for the dimensionality reduction to obtain 996 of the 1359 features before training the DNN-based DTI model. In the architecture of the DNN-based DTI model in Figure 3, the input layer had 996 nodes, the four hidden layers were separately embedded with 512, 256, 128 and 64 neurons, and the output layer had one node. We set the ReLU activation function for each hidden layer and the sigmoid activation function in the output layer. At the same time, the dropout layer was added to each hidden layer to prevent the DNN-based DTI model from overfitting issues during the training process. The DNN-based DTI model was applied with 5-fold cross-validation to evaluate the model’s performance, as shown in Table S5. The learning curves of loss and accuracy during the DNN-based DTI model training process are denoted in Figures S5 and S6, respectively. Eventually, the average test accuracy of 5-fold cross-validation is 93.06% with a standard deviation of 0.263%. The DNN-based DTI model with the best testing performance was utilized to predict the candidate drugs based on the probability of drug-target interactions of our identified drug targets. Additionally, we measured the well-trained DNN-based DTI model by the receiver operating characteristic (ROC) curves. The area under the curve (AUC) of the ROC is used to visualize the performance, as shown in Figure S7. The results show that the performance of the DNN-based DTI model (AUC = 0.980) is significantly better than that of the random prediction model (AUC = 0.5).

To further ensure the stability and safety of candidate drugs predicted by the DNN-based DTI model, we incorporated drug design specifications, such as regulation ability, sensitivity and toxicity. For the drug regulation ability, we downloaded the Library of Integrated Network-Based Cellular Signatures (LINCS) L1000 Level 5 dataset, which contains 12,328 genes treated with 19,811 small molecule compounds in 76 different human cell lines [81,82]. After the treatment with small-molecule compounds in LINCS L1000, we can determine whether the identified protein/gene expression is upregulated or downregulated, from which we can select potential molecule drugs from candidate drugs to restore the drug targets (biomarkers) to their normal expression. For drug sensitivity, we gathered the primary PRISM repurposing dataset [83], which included 4518 compounds in 578 human cell lines. We selected compounds with sensitivity values close to zero, which indicates that the cell line is insensitive to chemical perturbations. Furthermore, we considered drug toxicity (LC50) by using the ADMETlab 2.0 tool [84]. A drug with a higher LC50 value means it is less toxic to the body and usually has fewer side effects. Based on regulation ability, sensitivity and toxicity, some potential small-molecule compounds were selected for the identified drug targets of MIBC and ABC, as shown in Table 1. By considering the pharmacological properties, we selected the appropriate drug combinations as multiple-molecule drugs to satisfy the drug design specifications. Finally, we proposed the potential drug combination of Embelin and Obatoclax as the multiple-molecule drug to target three drug targets for the treatment of MIBC in Table 2; the potential drug combination of Obatoclax, Entinostat and Imiquimod as the multiple-molecule drug to target four drug targets for the treatment of ABC in Table 3.

## 3. Discussion

We investigated the specific and common core signaling pathways leading to the carcinogenic mechanism with the abnormal cellular functions of MIBC and ABC, as shown in Figure 2. We then identified the significant biomarkers of the oncogenic mechanisms of MIBC and ABC. Based on the well-trained DNN-based DTI model and drug-target interaction data, we could predict the interaction probabilities between candidate drugs and the identified drug targets. Subsequently, we proposed the potential molecular drug combinations as multiple-molecule drugs for the treatment of MIBC and ABC by considering drug design specifications, including regulation ability, sensitivity and toxicity, as shown in Table 2 and Table 3, respectively.

Among them, Obatoclax is a synthetic derivative of bacterial prodiginines [85], which was developed as a small-molecule antagonist of all of the antiapoptotic BCL-2 family members. A recent study showed that Obatoclax induced the downregulation of MYC and CCND1 and it might be the inhibitor of the WNT/β-catenin signaling pathway through downregulating LEF1 [86]. Another study revealed that Obatoclax could suppress cell proliferation and facilitate the effectiveness of cisplatin in MIBC cells [87]. Embelin (2,5-dihydroxy-3-undecyl-1,4-benzoquinone) is a small-molecule inhibitor of XIAP with antioxidant and anti-inflammatory activities in several human cancer cell lines. It has been documented that Embelin inhibits MYC, and might also be an inhibitor of nuclear factor-κB (NF-κB) [88]. Moreover, Embelin could inhibit invasion and cell proliferation, and induce apoptosis in mouse pancreatic cancer cells [89]. Entinostat (ENT) is a selective synthetic benzamide derivative histone deacetylase (HDAC) inhibitor that has been used in breast cancer to initiate apoptosis and inhibit cell proliferation [90]. It has been demonstrated that the ENT treatment increased the expression of FOXO1 in urothelial bladder cancer cells [91]. Furthermore, ENT downregulated MYC and the G2/M checkpoint in breast cancer [92]. Imiquimod is a small synthetic compound that typically treats skin cancer by activating an antitumor immune response [93]. The immunomodulatory treatment with imiquimod upregulated the expression of NOTCH1 in tumor cells [94]. In addition, the intravesical instillation of imiquimod has demonstrated reduced cell viability and apoptosis stimulation, resulting in reduced tumor growth and inflammatory response induction [95]. In summary, we separately proposed two molecular drug combinations: Embelin and Obatoclax as the multiple-molecule drug for MIBC; Obatoclax, Entinostat and Imiquimod as the multiple-molecule drug for ABC.

Cisplatin-based chemotherapy has been the standard therapy in major metastatic settings for the past decade, but the treatment paradigm for bladder cancer is rapidly changing as more and more relevant studies are discovered. Most recently, Erdafintib and Enfortumab vedotin (EV) have been approved for patients with advanced bladder cancer [2]. In this study, drug repurposing by applying the systematic drug design method is a promising option compared to the traditional drug design. It should be noted that the small-molecule compounds that we selected as drug combinations for the identified drug targets of MIBC and ABC were primarily U.S. Food and Drug Administration (FDA)-approved drugs and clinical trial drugs. The advantages of our systematic drug design and discovery are given as follows: First, repurposing FDA-approved drugs and clinical trial drugs can reduce the shortcomings in the traditional drug design process, especially the consumption of development time. Second, by choosing drug combinations as multiple-molecule drugs for multiple drug targets, the dose of monotherapy and drug toxicity can be reduced. Although further clinical research is required for validation, it is expected that patients with MIBC and ABC could benefit from systematic drug combination therapy.

## 4. Materials and Methods

### 4.1. Overview of Systems Biology Method and Systematic Drug Discovery and Design for MIBC and ABC

To comprehensively investigate the molecular carcinogenic mechanisms of MIBC and ABC, we utilized the systems biology approach [96] to construct the core GWGENs and analyze the common and specific core signaling pathways of MIBC and ABC. After identifying the significant biomarkers of oncogenic mechanisms as drug targets from the core signaling pathways, we designed a systematic drug discovery method based on the DNN-based DTI model and drug design specifications to sieve the potential drug combinations as multiple-molecule drugs for the treatment of MIBC and ABC. In this study, the flowcharts of the systems biology method and systematic drug discovery and design are shown in Figure 1 and Figure 3, respectively. Here, we divided the systems biology for the systematic drug discovery and design procedure into five steps:

(I). The construction of candidate GWGEN: We constructed the candidate GWGEN consisting of the candidate protein-protein interaction network (PPIN) and the candidate gene regulatory network (GRN) by big data mining.
(II). The identification of real GWGENs: The false-positive protein interactions and gene regulations in candidate GWGENs were pruned by the system identification scheme and system order detection method of Akaike information criterion (AIC) to obtain the real GWGENs for MIBC and ABC via the genome-wide microarray data downloaded from the National Center for Biotechnology Information (NCBI) GSE87304.
(III). The extraction of core GWGENs: From the perspective of network significance, the core GWGENs of MIBC and ABC were extracted by the PNP method from the real GWGENs.
(IV). The investigation of genetic and epigenetic oncogenic mechanisms: After identifying the core signaling pathways of MIBC and ABC based on the core GWGENs and the KEGG pathway annotations. We investigated the significant genetic and epigenetic oncogenic mechanisms in a microenvironment to distinguish the common and specific core signaling pathways between MIBC and ABC. Based on the oncogenic mechanisms, we selected the significant biomarkers from the core signaling pathways of MIBC and ABC as drug targets to interrupt bladder cancer progression and development.
(V). Potential drug combinations discovery and multiple-molecule drug design: The DNN-based DTI model was trained by the drug-target interaction databases. Since the DNN-based DTI model could precisely predict the interaction probability between drug targets and molecular drugs, we obtained candidate molecular drugs that can dock the drug targets of MIBC and ABC, respectively. Subsequently, we separately filtered the potential molecular drug combinations as multiple-molecule drugs of MIBC and ABC from the candidate molecular drugs, according to the drug design specifications, such as regulation ability, sensitivity and toxicity.

### 4.2. Data Preprocessing, Big Data Mining and the Construction of Candidate GWGEN

In this research, the genome-wide microarray dataset with accession number GSE87304 was downloaded from the Gene Expression Omnibus (GEO) of the NCBI (https://www.ncbi.nlm.nih.gov/geo/query/acc.cgi?acc=GSE87304, accessed on 6 November 2022). The corresponding experimental platform is GPL22995. The dataset included proteins, receptors, TFs, miRNAs and lncRNAs. Samples in the dataset were pre-NAC transurethral resection specimens. In general, MIBC is the patient with stage $T_{2}$ and ABC is the patient with stage $T_{3-4}$ [97]. After data preprocessing, we individually obtained 142 and 158 samples for MIBC and ABC on the basis of the clinical tumor stage. In addition, the candidate GWGEN in human cells consisted of the candidate protein-protein interactions (PPIs) as well as the regulations of genes, miRNAs and lncRNAs. The candidate GWGEN is composed of candidate PPIN and candidate GRN through big data mining. The candidate PPIN was based on the IntAct molecular interaction database [98], the Database of Interacting Proteins (DIP) [99], the Molecular INTeraction Database (MINT) [100], the Biomolecular Interaction Network Database (BIND) [101] and the Biological General Repository for Interaction Datasets database (BioGRID) [102]; the candidate GRN was based on the CircuitsDB database [103], the StarBase2.0 database [104], the TargetScanHuman database [105], the TRANScription FACtor database (TRANSFAC) [106], the Integrated Transcription Factor Platform database (ITFP) [107] and the Human Transcriptional Regulation Interactions database (HTRIdb) [108]. The candidate GWGEN was a boolean matrix. If there was an interaction between two nodes, we set the value to one; if there was no interaction, we set it to zero.

### 4.3. Systems Modeling for the Candidate GWGEN of MIBC and ABC

In order to identify the real GWGENs from candidate GWGEN by the corresponding genome-wide microarray data for investigating the molecular carcinogenic mechanisms of MIBC and ABC, we constructed the stochastic interactive and regulatory models of the candidate GWGEN, where the interactive model of proteins and the regulatory model of genes, miRNAs, and lncRNAs require the consideration of basal-level and stochastic noise due to model residues and data measurement noise.

For the protein interactive model, the s-th protein of the n-th sample are given by the following equation:

(1) $$p_{s}\left[n\right]=\sum_{\begin{array}{l} w=1\\ w\neq s \end{array}}^{E_{s}}\tau_{sw}p_{s}\left[n\right]p_{w}\left[n\right]+\zeta_{s,PPIN}+\psi_{s,PPIN}\left[n\right]$$

for s=1,…,S, n=1,…,N

where $p_{s}\left[n\right]$ and $p_{w}\left[n\right]$ indicate the expression level of the s-th and the w-th protein in the n-th sample; $\tau_{sw}$ represents the interaction ability between the s-th protein and the w-th protein; $E_{s}$ stands for the total number of proteins interacting with the s-th protein; S is the total number of proteins in the candidate PPIN; N is the total number of the data samples (patients); $\zeta_{s,PPIN}$ represents the basal level of the s-th protein expression due to unknown interactions of histone modifications, such as phosphorylation and acetylation; $\psi_{s,PPIN}\left[n\right]$ indicates the stochastic noise of the s-th protein in the n-th sample because of data measurement noise.

For the gene regulatory model, the transcriptional regulation of the t-th gene in the n-th sample is described by the following equation:

(2) $$g_{t}\left[n\right]=\sum_{f=1}^{F_{t}}\alpha_{tf}t_{f}\left[n\right]+\sum_{g=1}^{G_{t}}\beta_{tg}i_{g}\left[n\right]-\sum_{h=1}^{H_{t}}\delta_{th}m_{h}\left[n\right]g_{t}\left[n\right]+\zeta_{t}+\psi_{t}\left[n\right]$$

for t=1,…,T, n=1,…,N

where $g_{t}\left[n\right]$, $t_{f}\left[n\right]$, $i_{g}\left[n\right]$ and $m_{h}\left[n\right]$ denote the expression level of the t-th gene, the f-th TF, the g-th lncRNA and the h-th miRNA in the *n*-th sample, respectively; $\alpha_{tf}$ and $\beta_{tg}$ separately indicate the transcriptional regulatory ability of the f-th TF and the g-th lncRNA on the t-th gene; $\delta_{th}\geq 0$ is the post-transcriptional regulatory ability of the h-th miRNA to inhibit the t-th gene; $F_{t}$, $G_{t}$ and $H_{t}$ individually represent the total binding number of TFs, lncRNAs and miRNAs in the t-th gene; T is the total number of genes; N is the total number of the data samples (patients); $\zeta_{t}$ is the basal level of the t-th gene expression caused by unknown gene regulations, such as methylation; $\psi_{t}\left[n\right]$ is the stochastic noise of the t-th protein in the n-th sample including data noise. The system modeling and identification of lncRNA and miRNA is described in the Supplementary Materials.

### 4.4. The System Identification Scheme and System Order Detection Method for Real GWGENs of MIBC and ABC

In the previous section, we performed systems modeling for proteins, genes, miRNAs and lncRNAs in the candidate GWGEN. The MIBC and ABC shared the same candidate, GWGEN. However, the candidate GWGEN consists of all possible interactions and regulations derived from experimental and computational results, which include many false positives. Therefore, to prune the false positives in the candidate GWGEN, we performed the system identification scheme and system order detection method to separately obtain the real GWGENs of MIBC and ABC through their genome-wide microarray datasets.

To identify the interactive and regulatory parameters of the stochastic models, we need to solve the corresponding constrained least-square problems. Equations (1) and (2) can be individually rewritten in linear regression forms, as shown:

(3) $$\begin{array}{ll} p_{s}[n] & =\left[p_{s}[n]p_{1}[n]\cdots p_{s}[n]p_{E_{s}}[n]1\right]\times \left[\begin{array}{c} \;\tau_{s1}\\ \vdots \\ \;\tau_{sE_{s}}\\ \zeta_{s} \end{array}\right]+\psi_{s}\left[n\right]\\ & ≜\;\omega_{s}[n]\cdot \theta_{s}+\psi_{s}\left[n\right],\;\mathrm{for}\;s=1,\ldots ,S,\;n=1,\ldots ,N \end{array}$$

(4) $$\begin{array}{ll} g_{t}[n] & =\left[t_{1}[n]\;\cdots {\;t}_{F_{t}}[n]i_{1}[n]\;\cdots \;i_{G_{t}}[n]m_{1}[n]g_{t}[n]\;\cdots \;m_{H_{t}}[n]g_{t}[n]1\right]\times \left[\begin{array}{c} \;\alpha_{t1}\\ \vdots \\ \;\alpha_{tF_{t}}\\ \;\beta_{t1}\\ \vdots \\ \;\beta_{tG_{t}}\\ -\delta_{t1}\\ \vdots \\ -\delta_{tH_{t}}\\ \zeta_{t} \end{array}\right]+\psi_{t}\left[n\right]\;\\ & ≜\omega_{t}[n]\cdot \theta_{t}+\psi_{t}\left[n\right],\;\mathrm{for}\;t=1,\ldots ,T,\;n=1,\ldots ,N \end{array}$$

where $\omega_{s}[n]$ and $\omega_{t}[n]$ are the regression vector of the expression data for proteins and genes in the n-th sample, respectively; $\theta_{s}$ indicates the parameter vector of the protein-protein interaction abilities and basal levels of proteins; $\theta_{t}$ represents the parameter vector of the transcriptional regulatory abilities and basal levels of the genes; $\psi_{s}[n]$ and $\psi_{t}[n]$ individually denote the stochastic noise vectors of the s-th protein and the t-th gene in the n-th sample due to data noise.

Equations (3) and (4) extended to N samples are separately given by the following forms:

(5) $$\left[\begin{array}{c} p_{s}[1]\\ p_{s}[2]\\ \vdots \\ p_{s}[N] \end{array}\right]=\left[\begin{array}{c} \omega_{s}[1]\\ \omega_{s}[2]\\ \vdots \\ \omega_{s}[N] \end{array}\right]\cdot \theta_{s}+\left[\begin{array}{c} \psi_{s}\left[1\right]\\ \psi_{s}\left[2\right]\\ \vdots \\ \psi_{s}\left[N\right] \end{array}\right]$$

for s=1,…,S, n=1,…,N

(6) $$\left[\begin{array}{c} g_{t}[1]\\ g_{t}[2]\\ \vdots \\ g_{t}[N] \end{array}\right]=\left[\begin{array}{c} \omega_{t}[1]\\ \omega_{t}[2]\\ \vdots \\ \omega_{t}[N] \end{array}\right]\cdot \theta_{t}+\left[\begin{array}{c} \psi_{t}\left[1\right]\\ \psi_{t}\left[2\right]\\ \vdots \\ \psi_{t}\left[N\right] \end{array}\right]$$

for t=1,…,T, n=1,…,N

Equations (5) and (6) are simply represented by the following formulas:

(7) $$P_{s}=\Gamma_{s}\cdot \theta_{s}+\Xi_{s},\;\mathrm{for}\;s=1,\ldots ,S$$

(8) $$G_{t}=\Gamma_{t}\cdot \theta_{t}+\Xi_{t},\;\mathrm{for}\;t=1,\ldots ,T$$

To avoid overfitting problems in the system identification of the network construction procedure, the maximum degree of the parameter vector of proteins in PPINs and genes in GRNs must be less than half of the dataset samples [109]. Therefore, the parameter vectors $\theta_{s}$ and $\theta_{t}$ are estimated by solving the constrained linear least-squares parameter estimation problem via the MATLAB optimization toolbox as follows:

(9) $${\hat{\theta }}_{s}=\underset{\theta_{s}}{\arg\min}\frac{1}{2}{\left\|\Gamma_{s}\cdot \theta_{s}-P_{s}\right\|}_{2}^{2}$$

(10) $${\hat{\theta }}_{t}=\underset{\theta_{t}}{\arg\min}\frac{1}{2}{\left\|\Gamma_{t}\cdot \theta_{t}-G_{t}\right\|}_{2}^{2}$$

$$\begin{array}{l} \mathrm{subject}\;\mathrm{to}\;\left[\begin{array}{ccccccccccccc} 0 & \cdots & \cdots & 0 & 0 & \cdots & \cdots & 0 & 1 & 0 & \cdots & 0 & 0\\ \vdots & \ddots & & \vdots & \vdots & \ddots & & \vdots & 0 & \ddots & \ddots & \vdots & \vdots \\ \vdots & & \ddots & \vdots & \vdots & & \ddots & \vdots & \vdots & \ddots & \ddots & 0 & \vdots \\ 0 & \cdots & \cdots & 0 & 0 & \cdots & \cdots & 0 & 0 & \cdots & 0 & 1 & 0 \end{array}\right]\theta_{t}\leq \left[\begin{array}{c} 0\\ \begin{array}{l} \vdots \\ \vdots \end{array}\\ 0 \end{array}\right]\\ {\;\;\;\;\;\;\;\;\;\;\;\;\;\;\;\;\;\;\;\;\;\;\;\;\;\;\;\;\;\;\;\;\;\;\;\;\;F}_{t}{\;\;\;\;\;\;\;\;\;\;\;\;\;\;\;\;\;\;\;\;\;\;G}_{t}{\;\;\;\;\;\;\;\;\;\;\;\;\;\;\;\;\;\;\;\;\;\;\;H}_{t} \end{array}$$

In the meantime, the constraint condition on the least-squares parameter estimation problem in (10) means the estimated post-transcriptional regulatory abilities of miRNAs on genes are guaranteed to be negative.

The parameters of the candidate GWGENs of MIBC and ABC were estimated from the corresponding microarray data. Since the different experimental conditions may cause errors in the data from various databases, we utilized the Akaike information criterion (AIC) to prune the false positives and detect the system order (the number of interactions or regulations) of the real GWGENs. The equations of AIC for the s-th protein and the t-th gene can be described as follows:

(11) $$\begin{array}{l} AIC(E_{s})=\log({\hat{\Phi }}_{s}^{2})+\frac{2(E_{s}+1)}{N},\\ \mathrm{where}\;{\hat{\Phi }}_{s}=\sqrt{\frac{{\left(P_{s}-(\Gamma_{s}\cdot {\hat{\theta }}_{s})\right)}^{T}(P_{s}-(\Gamma_{s}\cdot {\hat{\theta }}_{s}))}{N}\;} \end{array}$$

where ${\hat{\Phi }}_{s}$ and $E_{s}$ separately denote the estimated residual error and the number (system order) of PPIs for the parameter estimation problem (9) in the s-th protein; ${\hat{\theta }}_{s}$ represents the estimated parameter vector for the s-th protein by (9).

(12) $$\begin{array}{l} AIC(F_{t},G_{t},H_{t})=\log({\hat{\Phi }}_{t}^{2})+\frac{2(\Omega_{t}+1)}{N},\\ \mathrm{where}\;{\hat{\Phi }}_{t}=\sqrt{\frac{{\left(G_{t}-(\Gamma_{t}\cdot {\hat{\theta }}_{t})\right)}^{T}(G_{t}-(\Gamma_{t}\cdot {\hat{\theta }}_{t}))}{N}\;},\Omega_{t}=F_{t}+G_{t}+H_{t} \end{array}$$

where ${\hat{\Phi }}_{t}$ and $\Omega_{t}$ individually indicate the estimated residual error and the number (system order) of regulations for the parameter estimation problem (10) in the t-th gene; ${\hat{\theta }}_{s}$ represents the estimated parameter vector for the t-th gene by (10).

According to the AIC system order detection method [109], the real system order of system modeling would minimize the AIC. To obtain the real system order in the candidate GWGEN, the AIC can be minimized by the following system order detection method:

(13) $$E_{s}^{*}=\underset{E_{s}}{\mathrm{argmin}}AIC(E_{s}),\;\mathrm{for}\;s=1,\ldots ,S$$

(14) $$F_{t}^{*},G_{t}^{*},H_{t}^{*}=\underset{F_{t},G_{t},H_{t}}{\mathrm{argmin}}AIC(F_{t},G_{t},H_{t}),\;\mathrm{for}\;t=1,\ldots ,T$$

where $E_{s}^{*}$ is the real number of PPIs for the s-th protein; $F_{t}^{*},G_{t}^{*},H_{t}^{*}$ denote the real number of regulations by TFs, lncRNAs and miRNAs on the t-th gene, respectively.

Consequently, the protein interactions and regulations left out of the real system order by the AIC in (13) and (14) are considered false positives, which would be pruned away to obtain the real GWGENs of MIBC and ABC. The method of performing system identification and utilizing the system order detection scheme for lncRNA and miRNA is given in the Supplementary Materials.

### 4.5. The Principal Network Projection (PNP) Method for Extracting the Core GWGENs from the Real GWGENs

After the system identification and system order detection methods, the real GWGENs of MIBC and ABC are still too complicated and difficult to investigate the genetic and epigenetic molecular mechanisms of carcinogenicity for MIBC and ABC. Hence, we applied the PNP method based on the singular value decomposition (SVD) to extract the core GWGENs from the relevant real GWGENs. In order to perform the PNP method, we have to construct a combined network matrix W that integrates all the estimated parameters in the real GWGENs as below:

(15) $$W=\left[\begin{array}{ccc} w_{protein↔protein} & 0 & 0\\ w_{TF\to gene} & w_{lncRNA\to gene} & w_{miRNA\to gene}\\ w_{TF\to lncRNA} & w_{lncRNA\to }{}_{lncRNA} & w_{miRNA\to lncRNA}\\ w_{TF\to miRNA} & w_{lncRNA\to }{}_{miRNA} & w_{miRNA\to miRNA} \end{array}\right]$$

where the subnetwork matrix $w_{protein↔protein}$ is the system matrix of the estimated protein interaction abilities, the bidirectional arrow shows that the protein interaction is bidirectional; the subnetwork matrices $w_{TF\to gene}$, $w_{lncRNA\to gene}$ and $w_{miRNA\to gene}$ separately indicate the system matrices of the estimated transcriptional regulatory abilities for TFs, lncRNAs and miRNAs on genes; the subnetwork matrices $w_{TF\to lncRNA}$, $w_{lncRNA\to lncRNA}$ and $w_{miRNA\to lncRNA}$ individually denote the system matrices of the estimated transcriptional regulatory abilities for TFs, lncRNAs and miRNAs on lncRNAs; the subnetwork matrices $w_{TF\to miRNA}$, $w_{lncRNA\to miRNA}$ and $w_{miRNA\to miRNA}$ separately indicate the system matrices of the estimated transcriptional regulatory abilities for TFs, lncRNAs and miRNAs on miRNAs. The detailed network matrix W of the real GWGENs is described as follows:

(16) $$W=\left[\begin{array}{ccccccccccccccc} {\hat{\tau }}_{11} & \cdots & {\hat{\tau }}_{1w} & \cdots & {\hat{\tau }}_{1W} & 0 & \cdots & 0 & \cdots & 0 & 0 & \cdots & 0 & \cdots & 0\\ \vdots & \ddots & \vdots & \ddots & \vdots & \vdots & \ddots & \vdots & \ddots & \vdots & \vdots & \ddots & \vdots & \ddots & \vdots \\ {\hat{\tau }}_{s1} & \cdots & {\hat{\tau }}_{sw} & \cdots & {\hat{\tau }}_{sW} & 0 & \cdots & 0 & \cdots & 0 & 0 & \cdots & 0 & \cdots & 0\\ \vdots & \ddots & \vdots & \ddots & \vdots & \vdots & \ddots & \vdots & \ddots & \vdots & \vdots & \ddots & \vdots & \ddots & \vdots \\ {\hat{\tau }}_{S1} & \cdots & {\hat{\tau }}_{Sw} & \cdots & {\hat{\tau }}_{SW} & 0 & \cdots & 0 & \cdots & 0 & 0 & \cdots & 0 & \cdots & 0\\ {\hat{\alpha }}_{11} & \cdots & {\hat{\alpha }}_{1f} & \cdots & {\hat{\alpha }}_{1F} & {\hat{\beta }}_{11} & \cdots & {\hat{\beta }}_{1g} & \cdots & {\hat{\beta }}_{1G} & -{\hat{\delta }}_{11} & \cdots & -{\hat{\delta }}_{1h} & \cdots & -{\hat{\delta }}_{1H}\\ \vdots & \ddots & \vdots & \ddots & \vdots & \vdots & \ddots & \vdots & \ddots & \vdots & \vdots & \ddots & \vdots & \ddots & \vdots \\ {\hat{\alpha }}_{t1} & \cdots & {\hat{\alpha }}_{tf} & \cdots & {\hat{\alpha }}_{tF} & {\hat{\beta }}_{t1} & \cdots & {\hat{\beta }}_{tg} & \cdots & {\hat{\beta }}_{tG} & -{\hat{\delta }}_{t1} & \cdots & -{\hat{\delta }}_{th} & \cdots & -{\hat{\delta }}_{tH}\\ \vdots & \ddots & \vdots & \ddots & \vdots & \vdots & \ddots & \vdots & \ddots & \vdots & \vdots & \ddots & \vdots & \ddots & \vdots \\ {\hat{\alpha }}_{T1} & \cdots & {\hat{\alpha }}_{Tf} & \cdots & {\hat{\alpha }}_{TF} & {\hat{\beta }}_{T1} & \cdots & {\hat{\beta }}_{Tg} & \cdots & {\hat{\beta }}_{TG} & -{\hat{\delta }}_{T1} & \cdots & -{\hat{\delta }}_{Th} & \cdots & -{\hat{\delta }}_{TH}\\ {\hat{\varepsilon }}_{11} & \cdots & {\hat{\varepsilon }}_{1f} & \cdots & {\hat{\varepsilon }}_{1F} & {\hat{\gamma }}_{11} & \cdots & {\hat{\gamma }}_{1g} & \cdots & {\hat{\gamma }}_{1G} & -{\hat{\sigma }}_{11} & \cdots & -{\hat{\sigma }}_{1h} & \cdots & -{\hat{\sigma }}_{1H}\\ \vdots & \ddots & \vdots & \ddots & \vdots & \vdots & \ddots & \vdots & \ddots & \vdots & \vdots & \ddots & \vdots & \ddots & \vdots \\ {\hat{\varepsilon }}_{u1} & \cdots & {\hat{\varepsilon }}_{uf} & \cdots & {\hat{\varepsilon }}_{uF} & {\hat{\gamma }}_{u1} & \cdots & {\hat{\gamma }}_{ug} & \cdots & {\hat{\gamma }}_{uG} & -{\hat{\sigma }}_{u1} & \cdots & -{\hat{\sigma }}_{uh} & \cdots & -{\hat{\sigma }}_{uH}\\ \vdots & \ddots & \vdots & \ddots & \vdots & \vdots & \ddots & \vdots & \ddots & \vdots & \vdots & \ddots & \vdots & \ddots & \vdots \\ {\hat{\varepsilon }}_{U1} & \cdots & {\hat{\varepsilon }}_{Uf} & \cdots & {\hat{\varepsilon }}_{UF} & {\hat{\gamma }}_{U1} & \cdots & {\hat{\gamma }}_{Ug} & \cdots & {\hat{\gamma }}_{UG} & -{\hat{\sigma }}_{U1} & \cdots & -{\hat{\sigma }}_{Uh} & \cdots & -{\hat{\sigma }}_{UH}\\ {\hat{\phi }}_{11} & \cdots & {\hat{\phi }}_{1f} & \cdots & {\hat{\phi }}_{1F} & {\hat{\lambda }}_{11} & \cdots & {\hat{\lambda }}_{1g} & \cdots & {\hat{\lambda }}_{1G} & -{\hat{\mu }}_{11} & \cdots & -{\hat{\mu }}_{1h} & \cdots & -{\hat{\mu }}_{1H}\\ \vdots & \ddots & \vdots & \ddots & \vdots & \vdots & \ddots & \vdots & \ddots & \vdots & \vdots & \ddots & \vdots & \ddots & \vdots \\ {\hat{\phi }}_{v1} & \cdots & {\hat{\phi }}_{vf} & \cdots & {\hat{\phi }}_{vF} & {\hat{\lambda }}_{v1} & \cdots & {\hat{\lambda }}_{vg} & \cdots & {\hat{\lambda }}_{vG} & -{\hat{\mu }}_{v1} & \cdots & -{\hat{\mu }}_{vh} & \cdots & -{\hat{\mu }}_{vH}\\ \vdots & \ddots & \vdots & \ddots & \vdots & \vdots & \ddots & \vdots & \ddots & \vdots & \vdots & \ddots & \vdots & \ddots & \vdots \\ {\hat{\phi }}_{V1} & \cdots & {\hat{\phi }}_{Vf} & \cdots & {\hat{\phi }}_{VF} & {\hat{\lambda }}_{V1} & \cdots & {\hat{\lambda }}_{Vg} & \cdots & {\hat{\lambda }}_{VG} & -{\hat{\mu }}_{V1} & \cdots & -{\hat{\mu }}_{Vh} & \cdots & -{\hat{\mu }}_{VH} \end{array}\right]\in {\mathbb{R}}^{\left(S^{*}+T^{*}+U^{*}+V^{*})\times (F^{*}+G^{*}+H^{*}\right)}$$

where the zeros displayed in the component of the network matrix W represent no interaction or regulation after being pruned by the AIC. The network matrix W in (16) was decomposed by the PNP method on the basis of SVD as follows:

(17) $$W=U\Sigma V^{T}$$

where $U\in {\mathbb{R}}^{\left(S^{*}+T^{*}+U^{*}+V^{*})\times (S^{*}+T^{*}+U^{*}+V^{*}\right)}$ and $V\in {\mathbb{R}}^{\left(F^{*}+G^{*}+H^{*})\times (F^{*}+G^{*}+H^{*}\right)}$ indicate the unitary singular matrices; $\Sigma =diag(\Sigma_{1},\cdots ,\Sigma_{x},\cdots ,\Sigma_{F^{*}+G^{*}+H^{*}})\in {\mathbb{R}}^{\left(S^{*}+T^{*}+U^{*}+V^{*})\times (F^{*}+G^{*}+H^{*}\right)}$ is the diagonal matrix which contains $F^{*}+G^{*}+H^{*}$ singular values of the matrix W in descending order (i.e., $\Sigma_{1}\geq \cdots \geq \Sigma_{x}\geq \cdots \geq \Sigma_{F^{*}+G^{*}+H^{*}}\geq 0$). The diagonal matrix Σ is shown as below:

(18) $$\Sigma =\left[\begin{array}{ccccc} \Sigma_{1} & \cdots & 0 & \cdots & 0\\ \vdots & \ddots & \vdots & \ddots & \vdots \\ 0 & \cdots & \Sigma_{x} & \cdots & 0\\ \vdots & \ddots & \vdots & \ddots & \vdots \\ 0 & \cdots & 0 & \cdots & \Sigma_{F^{*}+G^{*}+H^{*}}\\ 0 & \cdots & 0 & \cdots & 0\\ \vdots & \ddots & \vdots & \ddots & \vdots \\ 0 & \cdots & 0 & \cdots & 0 \end{array}\right]\in {\mathbb{R}}^{\left(S^{*}+T^{*}+U^{*}+V^{*})+(F^{*}+G^{*}+H^{*}\right)}$$

Furthermore, we defined the eigen expression fraction $E_{x}$ of the normalized singular values in (18) as follows:

(19) $$E_{x}=\frac{\Sigma_{x}^{2}}{\sum_{x=1}^{F^{*}+G^{*}+H^{*}}\Sigma_{x}^{2}}\;,\sum_{x=1}^{F^{*}+G^{*}+H^{*}}E_{x}=1$$

From the perspective of energy, we selected the top X singular vectors of the matrix W with energy greater than or equal to the threshold of 0.85 in (20).

(20) $$\sum_{x=1}^{X}E_{x}\geq 0.85$$

The top X principal singular vectors denote the principal network structure with 85% (principal) energy of the real GWGENs. Subsequently, we individually projected each node of the real GWGENs (i.e., each row of the matrix W) to the top X principal singular vectors in the following:

(21) $$\begin{array}{l} P(a,b)=w_{a,:}\cdot v_{:,b}^{T}\;,\\ \mathrm{for}\;a=1,\ldots ,S^{*}+T^{*}+U^{*}+V^{*},\;b=1,\ldots ,X \end{array}$$

where P(a,b) represents the projection value of the a-th node to the b-th principal singular vector; $w_{a,:}$ and $v_{:,b}$ indicate the a-th row vector and the b-th principal singular vector of the matrix W, respectively. Then, we define the 2-norm projection value of each node, such as protein, gene, miRNA and lncRNA in the real GWGENs to the top X principal singular vectors as follows:

(22) $$\begin{array}{l} Q(a)=\sqrt{\sum_{b=1}^{X}P^{2}(a,b)}\;,\\ \mathrm{for}\;a=1,\ldots ,S^{*}+T^{*}+U^{*}+V^{*},\;b=1,\ldots ,X \end{array}$$

where Q(a) represents the 2-norm projection value of the a-th node at the top X principal singular vectors. The larger the projection value Q(a) in the real GWGENs, the more significant the corresponding a-th node to the principal network structure. On the contrary, the projection value Q(a) is close to zero, the corresponding a-th node indicates insignificance and almost independence from the principal network structure.

The core GWGENs of MIBC and ABC were extracted from the real GWGENs by the top 6000 projection values Q(a), respectively, i.e., core GWGENs contain 6000 significant nodes, which are the maximum number allowed for KEGG pathway enrichment analysis and pathway annotations in the following. Next, we separately uploaded the nodes of the core GWGENs of MIBC and ABC to the DAVID website for KEGG pathway enrichment analysis and obtained the core signaling pathways of MIBC and ABC by exploring the KEGG pathway annotations and literature survey. Ultimately, we compared the core signaling pathways of MIBC and ABC to investigate the genetic and epigenetic carcinogenic mechanisms between them. From the carcinogenic mechanisms of MIBC and ABC, we chose the essential biomarkers as their drug targets in Table 1.

### 4.6. Systematic Discovery and Design of Drug Combinations as Multiple-molecule Drugs for MIBC and ABC via Deep Neural Network

We proposed a DNN-based DTI model to predict the interaction probability between the drugs and targets (biomarkers) and specified the drug design specifications to sieve the potential drug combinations as multiple-molecule drugs for the drug targets of MIBC and ABC, respectively. The flowchart of systems drug discovery and design for the treatment of MIBC and ABC is shown in Figure 3.

In order to train the DNN-based DTI model for predicting candidate drugs for the drug targets, we integrated the drug-target interaction databases from STITCH [110], BIDD [111], UniProt [112], DrugBank [113], ChEMBL [114], PubChem [115] and KEGG [116], which contain information on molecular docking and the features of drugs and genes. The drug features include constitutional, topological, geometrical descriptors and other molecular properties. The sequence of the target protein is representative because the complete information of the target protein is generally considered to be encoded in the sequence. The target features were calculated from the structural and physicochemical features of proteins and peptides in the amino acid sequences. After transforming the properties of drugs and targets into feature vectors with the Python package PyBioMed under the Python 3.7 environment [117], each drug-target pair is represented by concatenating the corresponding feature vectors. The feature vector of the drug-target pair is given as follows:

(23) $$z_{\mathrm{drug}-\mathrm{target}}=\left[D,T\right]=\left[d_{1},\cdots ,d_{M},t_{1},\cdots ,t_{N}\right]$$

where $z_{\mathrm{drug}-\mathrm{target}}$ is the feature vector of the drug-target pair; D indicates the feature vector of the corresponding drug; T indicates the feature vector of the relevant target; $d_{m}$ denotes the m-th drug feature; $t_{n}$ denotes the n-th target feature; M is the total number of drug features; N is the total number of target features.

Before employing the drug-target feature vectors as training data for the DTI model, we preprocessed the training data to avoid the problem of possible bias in the original feature values. First, since the unproven interaction data (i.e., negative class) were much larger than the proven interaction data (i.e., positive class), the number of the negative class needed to be randomly down-sampled to the number of the positive class to prevent the biased parameter updates for larger classes during model training. Second, because the values of the feature vectors in each drug-target pair are measured in various units, it may cause numerical precision errors and degrade the performance of the DTI model. For the feature scaling problem, the min-max normalization and standardization methods are typically applied to solve the problem. While the min-max normalization can normalize the data, it is more sensitive to outliers than standardization. Therefore, we applied the standardization to the features of each drug and target, as shown below:

(24) $$d_{i}^{*}=\frac{d_{i}-\mu_{i}}{\sigma_{i}},\;\forall i=1,\cdots ,M$$

(25) $$t_{j}^{*}=\frac{t_{j}-\mu_{j}}{\sigma_{j}},\;\forall j=1,\cdots ,N$$

where $d_{i}^{*}$ denotes the i-th drug feature after standardization; $d_{i}$ denotes the i-th drug feature; $\mu_{i}$ and $\sigma_{i}$ separately the stand for the mean and standard deviation of the i-th drug feature; $t_{j}^{*}$ denotes the j-th target feature after standardization; $t_{j}$ denotes the j-th target feature; $\mu_{j}$ and $\sigma_{j}$ individually represent the mean and standard deviation of the j-th target feature; M is the total number of drug features; N is the total number of target features.

Since the dimension of the training data (feature vector) is too high, it might increase the number of features in the DNN and the computational complexity of model training. We utilized the principal component analysis (PCA) method to reduce the dimension of the feature vector [118] after down-sampling and standardization. Subsequently, we trained the DTI model based on DNN to predict drug-target interaction through the Tensorflow and Keras packages. After preprocessing the data, we used three-quarters of the data as training data and one-quarter of the data as testing data. Furthermore, the DNN-based DTI model has 996 nodes as the input layer and four hidden layers with 512, 256, 128 and 64 neurons, respectively, and each hidden layer has a rectified linear unit (ReLU) activation function to avoid the vanishing gradient problem in deep learning. The activation function of the output layer uses the sigmoid function, which can restrict the output value in the range of 0–1 as the model prediction probability. In the architecture of the DNN-based DTI model, the equation for each layer can be described as follows:

(26) $$h_{n}=\sigma (wx_{n}+b)$$

where $h_{n}$ and $x_{n}$ indicate the output and the input of the n-th drug-target feature vector, respectively; σ is the activation function of the ReLU function in the hidden layer and the sigmoid function in the output layer; w denotes the weighting matrix; b indicates the bias vector. Due to the drug-target interaction being a binary classification issue, we chose the binary cross-entropy as the cost function to calculate the model loss, as shown below:

(27) $$C_{n}(p_{n},\;{\hat{p}}_{n})=-[p_{n}\log{\hat{p}}_{n}+(1-p_{n})log(1-{\hat{p}}_{n})]$$

(28) $$L(w,b)=\frac{1}{N}\sum_{n=1}^{N}C_{n}(w,b)$$

where $p_{n}$ is the true probability of positive interaction in the n-th sample; ${\hat{p}}_{n}$ is the predicted probability of positive interaction in the n-th sample; $1-p_{n}$ denotes the true probability of negative interaction in the n-th sample, $1-{\hat{p}}_{n}$ denotes the predicted probability of negative interaction in the n-th sample; L(w,b) represents the average of the total loss $C(p_{n},{\hat{p}}_{n})$; N represents the total number of the training data.

On the basis of the cost function, we applied the backward propagation algorithm [119] to update the model parameter set θ of the weight matrix w and bias vector b by calculating the gradient to obtain the optimal model parameter set $\theta^{*}$. The gradient iterative algorithm is described as follows:

(29) $$\theta =\left[\begin{array}{c} w\\ b \end{array}\right]$$

(30) $$\theta^{*}=\underset{\theta }{\arg\min}\;L(\theta )$$

(31) $$\begin{array}{l} \theta^{z}=\theta^{z-1}-\eta \nabla L(\theta^{z-1}),\;\\ \mathrm{where}\;\nabla L(\theta^{z-1})=\left[\begin{array}{c} \frac{\partial L(\theta^{z-1})}{\partial w}\\ \frac{\partial L(\theta^{z-1})}{\partial b} \end{array}\right] \end{array}$$

where z indicates the z-th iteration of the DNN learning process; η represents the learning rate; $\nabla L(\theta^{z-1})$ stands for the gradient of $L(\theta^{z-1})$.

The backward propagation method can efficiently calculate the high-dimensional vector $\nabla L(\theta^{z-1})$ and adapt the DNN-based DTI model parameters to fit the drug-target interaction data well for each iteration. Moreover, the adjustment of the hyperparameters can attain the optimal model performance, we trained the DNN-based DTI model by setting the learning rate as 0.001 under the Python 3.7 environment. Then, we also set epochs to 100 and batch size to 100. To verify the stability and prediction performance of the DNN-based DTI model during the training process, we applied the 5-fold cross-validation method to the training data. We divided the training data into five equal parts and took one of them as the validation data each time, and the remaining data were used as the training data until each part was used as the validation data, as shown in Figures S5 and S6. We also employed the early stopping approach to prevent the problem of model training overfitting. After completing the training of the DNN-based DTI model, we adopted the area under the curve (AUC) and receiver operating characteristic (ROC) curves [120] of the performance measurement to examine the performance of the DNN-based DTI model. For classification problems, it is one of the most helpful evaluation metrics for visualizing model performance. The larger the area under the line, the higher the AUC and the higher the accuracy of the DNN-based DTI model in predicting true-positive and true-negative drug-target interactions. The equations of the AUC and ROC curves are described as follows:

(32) $$\mathrm{TPR}(\mathrm{True}\;\mathrm{Positive}\;\mathrm{Rate})=\frac{\mathrm{TP}}{\mathrm{TP}+\mathrm{FN}}$$

(33) $$\mathrm{specificity}=\frac{\mathrm{TN}}{\mathrm{TN}+\mathrm{FP}}$$

(34) $$\mathrm{FPR}(\mathrm{False}\;\mathrm{Positive}\;\mathrm{Rate})=1-\mathrm{specificity}=\frac{\mathrm{FP}}{\mathrm{TN}+\mathrm{FP}}$$

where TP (True Positive) denotes the probability that the real value is true and is judged accurately; TN (True Negative) denotes the probability that the real value is true and is judged in error; FP (False Positive) is the probability that the real value is false and is judged correctly; FN (False Negative) is the probability that the real value is false and is judged incorrectly.

The steps to discover drug combinations from candidate molecules as multiple-molecule drugs based on drug design specifications for MIBC and ABC are as follows: First, we considered the regulation ability of the candidate drugs, which were predicted by the DNN-based DTI model. We integrated the regulation ability data of the candidate drugs from the library of integrated network-based cellular signatures (LINCS) in the L1000 Level 5 dataset [81], the positive value indicates the upregulation and the negative value means the downregulation. Thus, if the gene expression of the selected biomarker (drug target) is of abnormal upregulation, we would select the drugs with a negative correlation. In contrast, if the gene expression of the chosen biomarker (drug target) is of abnormal downregulation, the drugs with a positive correlation will be chosen. Afterward, we checked the drug sensitivity and the corresponding dataset obtained from primary PRISM repurposing datasets [83]. We selected the compounds with sensitivity values close to zero, which denote that the cell line is insensitive to chemical perturbations. Lastly, we also considered the toxicity of the drugs because they could have negative effects on health. The drug toxicity (LC50) is regularly considered for cancer drug design. We referred to the LC50 value by using the ADMETlab 2.0 tool [84], the higher the LD50 value, the lower the toxicity. Finally, we individually proposed the potential molecular drug combinations as multiple-molecule drugs for the drug targets of MIBC and ABC through the screening of the drug design specifications.

## 5. Conclusions

We explored the differences in carcinogenic mechanisms between MIBC and ABC from a systems biology perspective through systems modeling based on the systematic identification methodology with their genome-wide microarray data. A better understanding of the genetic and epigenetic molecular mechanisms of MIBC and ABC leads to a more correct selection of their significant biomarkers as drug targets, thereby facilitating the development of novel potential therapeutics. Furthermore, with the help of the DNN-based DTI model to precisely predict the candidate drugs having interactions with the drug targets, we applied the concepts of engineering design specifications to establish systematic drug design and discovery for a more systematic view of the chemical and therapeutic relationships, such as regulation ability, toxicity, and sensitivity between drugs and drug targets.

In this study, based on the proposed systems biology methods, we selected NFKB1, LEF1, and MYC as drug targets for MIBC, and selected LEF1, MYC, NOTCH1, and FOXO1 as drug targets for ABC. Then, based on the proposed systematic drug design method, the potential drug combination of Embelin and Obatoclax was selected as the multiple-molecule drug to target multiple drug targets in MIBC, and Obatoclax, Entinostat, and Imiquimod were selected as the multiple-molecule drug to target multiple drug targets in ABC. With more biological and clinical validation, it is hoped that this study of systematic drug design will be beneficial to patients with MIBC and ABC.

## Figures and Tables

**Table 1:** Potential small-molecule compounds for each identified drug target of MIBC and ABC are selected from their candidate small-molecule compounds based on the drug design specifications.

**NFKB1 (+)**			
Candidate drugs	Regulation Ability (L1000)	Sensitivity (PRISM)	Toxicity (LC50, log(mol/kg))
Evodiamine	−0.453	−2.766	4.402
Embelin	−0.450	−0.563	5.223
Norethisterone	−0.448	0.325	2.946
Carbachol	−0.445	0.337	1.963
Zaleplon	−0.440	−1.522	3.324
**MYC (+)**			
Candidate drugs	Regulation Ability (L1000)	Sensitivity (PRISM)	Toxicity (LC50, log(mol/kg))
Embelin	−0.822	−0.563	5.223
Dexrazoxane	−0.671	−1.558	3.004
Obatoclax	−0.539	−2.694	5.255
Roquinimex	−0.526	−1.831	4.524
Entinostat	−0.525	−1.013	4.971
**LEF1 (+)**			
Candidate drugs	Regulation Ability (L1000)	Sensitivity (PRISM)	Toxicity (LC50, log(mol/kg))
Bortezomib	−0.711	−5.840	2.474
Navitoclax	−0.697	−0.913	3.97
Obatoclax	−0.678	−2.694	5.255
Mitomycin-c	−0.649	−1.269	3.017
Halofantrine	−0.625	−1.604	6.478
**FOXO1 (−)**			
Candidate drugs	Regulation Ability (L1000)	Sensitivity (PRISM)	Toxicity (LC50, log(mol/kg))
Domperidone	0.268	−1.109	4.148
Mevastatin	0.262	−2.724	3.481
Ispinesib	0.252	−2.887	5.70
Entinostat	0.235	−1.013	4.971
Acebutolol	0.212	0.722	2.778
**NOTCH1 (−)**			
Candidate drugs	Regulation Ability (L1000)	Sensitivity (PRISM)	Toxicity (LC50, log(mol/kg))
Tomelukast	0.942	0.077	3.192
Imiquimod	0.896	0.083	4.147
Quinelorane	0.887	0.294	2.850
Mephenesin	0.866	0.327	2.330
Celiprolol	0.847	0.140	2.590

NOTE: The small-molecule compounds with the gray background are the selected drugs for drug combinations as multiple-molecule drugs for MIBC in Table 2 and ABC in Table 3.

**Table 2:** The selected molecular drugs and their corresponding drug targets for the drug combination as the multiple-molecule drug for the treatment of MIBC.

Drug Names	Regulation Ability with Binding to Drug Targets		
	NFKB1	MYC	LEF1
Embelin	●	●	
Obatoclax		●	●
Chemical structures of molecular drug combination			
Embelin		Obatoclax	
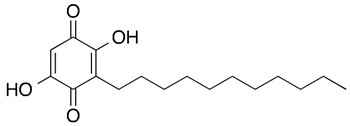		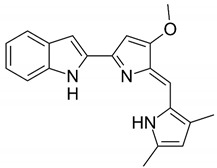	

●: The biomarker targeted by the corresponding molecule compounds.

**Table 3:** The selected molecular drugs and their corresponding drug targets for the drug combination as the multiple-molecule drug for the treatment of ABC.

Drug Names			Regulation Ability with Binding to Drug Targets											
			LEF1			MYC			FOXO1			NOTCH1		
Obatoclax			●			●								
Entinostat						●			●					
Imiquimod												●		
Chemical structures of molecular drug combination														
Obatoclax					Entinostat					Imiquimod				
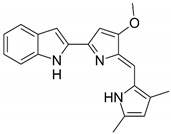					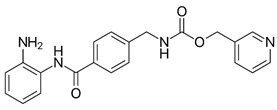					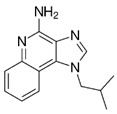				

●: The biomarker targeted by the corresponding molecule compounds.

## Data Availability

The muscle-invasive bladder cancer and the advanced bladder cancer microarray data come from GSE87304 (https://www.ncbi.nlm.nih.gov/geo/query/acc.cgi?acc=GSE87304) (accessed on 30 June 2021).

## Supplementary Materials

The following supporting information can be downloaded at: https://www.mdpi.com/article/10.3390/ijms232213869/s1.
