# Supplementary material for: Systems Drug Design for Muscle Invasive Bladder Cancer and Advanced Bladder Cancer by Genome-Wide Microarray Data and Deep Learning Method with Drug Design Specifications
Source: Int J Mol Sci. 2022 Nov 10;23(22):13869. doi: 10.3390/ijms232213869 (PMC9692470; doi:10.3390/ijms232213869)
Supplement: Supplementary file 1 [file ijms-23-13869-s001.zip › ijms-1928332-supplementary.pdf]

Supplementary Materials

S.1.1 Systems Modeling for the Candidate GWGEN of MIBC and ABC

The regulations of TFs, lncRNAs and miRNAs have a great effect on the expression level of the u-th lncRNA. For the candidate lncRNA regulatory model of the candidate GWGEN, the u-th lncRNA of the n-th sample can be described by the regulatory equation as follows:

$$l_u[n]=\sum_{f=1}^{F_u}\varepsilon_{uf}t_f[n]+\sum_{\substack{g=1\\g\neq u}}^{G_u}\gamma_{ug}i_g[n]-\sum_{h=1}^{H_u}\sigma_{uh}m_h[n]l_u[n]+\zeta_u+\psi_u[n] \tag{S1}$$

$$\text{for } u=1,\ldots,U,\ n=1,\ldots,N$$

where $l_u[n]$, $t_f[n]$, $i_g[n]$, $m_h[n]$ denote the expression level of the u-th lncRNA, the f-th TF, the g-th lncRNA, and the h-th miRNA in the *n*-th sample, respectively; ε_{uf} and γ_{ug} separately indicate the transcriptional regulatory ability of the f-th TF and the g-th lncRNA on the u-th lncRNA; $\sigma_{uh}\geq 0$ is the post-transcriptional regulatory ability of the h-th miRNA to inhibit the u-th lncRNA; F_u, G_u, H_u individually represent the total binding number of TFs, lncRNAs and miRNAs to the u-th lncRNA; U is the total number of lncRNAs; N is the total number of the data samples (patients); ζ_u means the basal level of the u-th lncRNA expression due to unknown regulations; $\psi_u[n]$ means the stochastic noise of the u-th lncRNA in the n-th sample including data noise.

Similarly, the regulations of TFs, lncRNAs and miRNAs also affect the expression level of the v-th miRNA. For the candidate miRNA regulatory model of the candidate GWGEN, the v-th miRNA of the n-th sample can be represented by the following regulatory equations:

$$c_v[n]=\sum_{f=1}^{F_v}\phi_{vf}t_f[n]+\sum_{g=1}^{G_v}\lambda_{vg}i_g[n]-\sum_{\substack{h=1\\h\neq v}}^{H_v}\mu_{vh}m_h[n]c_v[n]+\zeta_v+\psi_v[n] \tag{S2}$$

$$\text{for } v=1,\ldots,V,\ n=1,\ldots,N$$

where $c_v[n]$, $t_f[n]$, $i_g[n]$, $m_h[n]$ denote the expression level of the v-th miRNA, the f-th TF, the g-th lncRNA, and the h-th miRNA in the *n*-th sample, respectively; ϕ_{vf} and λ_{vg} separately indicate the transcriptional regulatory ability of the f-th TF and the g-th lncRNA on the v-th miRNA; $\mu_{vh}\geq 0$ is the post-transcriptional regulatory ability of the h-th miRNA on the v-th miRNA; F_v, G_v, H_v individually represent the total binding number of TFs, lncRNAs and miRNAs to the v-th miRNA; V is the total number of miRNAs; N is the total number of the data samples (patients); ζ_v means the basal level of the v-th miRNA expression due to unknown regulations; $\psi_v[n]$ means the stochastic noise of the v-th miRNA in the n-th sample including data noise.

S.1.2 The System Identification Scheme and System Order Detection Method for Real GWGENs of MIBC and ABC

To identify the interactive and regulatory parameters of the stochastic models, we need to solve the corresponding constrained least-square problems. The equations (S1) and (S2) can individually be rewritten by the linear regression forms in the following:

$$l_u[n] = \begin{bmatrix} t_1[n] & \cdots & t_{F_u}[n] & i_1[n] & \cdots & i_{G_u}[n] & m_1[n]l_u[n] & \cdots & m_{H_u}[n]l_u[n] & 1 \end{bmatrix} \times \begin{bmatrix} \varepsilon_{u1} \\ \vdots \\ \varepsilon_{uF_u} \\ \gamma_{u1} \\ \vdots \\ \gamma_{uG_u} \\ -\sigma_{u1} \\ \vdots \\ -\sigma_{uH_u} \\ \zeta_u \end{bmatrix} + \psi_u[n] \tag{S3}$$

$$\triangleq \omega_u[n] \cdot \theta_u + \psi_u[n], \text{ for } u = 1, \ldots, U, \ n = 1, \ldots, N$$

$$c_v[n] = \begin{bmatrix} t_1[n] & \cdots & t_{F_v}[n] & i_1[n] & \cdots & i_{G_v}[n] & m_1[n]c_v[n] & \cdots & m_{H_v}[n]c_v[n] & 1 \end{bmatrix} \times \begin{bmatrix} \phi_{v1} \\ \vdots \\ \phi_{vF_v} \\ \lambda_{v1} \\ \vdots \\ \lambda_{vG_v} \\ -\mu_{v1} \\ \vdots \\ -\mu_{vH_v} \\ \zeta_v \end{bmatrix} + \psi_v[n] \tag{S4}$$

$$\triangleq \omega_v[n] \cdot \theta_v + \psi_v[n], \text{ for } v = 1, \ldots, V, \ n = 1, \ldots, N$$

where $\omega_u[n]$ and $\omega_v[n]$ are the regression vector of the expression data for lncRNAs and miRNAs in the n-th sample, respectively; θ_u and θ_v separately represent the parameter vector of the transcriptional regulatory abilities and basal levels of lncRNAs and miRNAs; $\psi_u[n]$ and $\psi_v[n]$ individually denote the stochastic noise vectors of the u-th lncRNA and the v-th miRNA in the n-th sample due to data noise.

The equations (S3) and (S4) extended to N samples are separately given by the following forms:

$$\begin{bmatrix} l_u[1] \\ l_u[2] \\ \vdots \\ l_u[\mathrm{N}] \end{bmatrix} = \begin{bmatrix} \omega_u[1] \\ \omega_u[2] \\ \vdots \\ \omega_u[\mathrm{N}] \end{bmatrix} \cdot \theta_u + \begin{bmatrix} \psi_u[1] \\ \psi_u[2] \\ \vdots \\ \psi_u[N] \end{bmatrix} \tag{S5}$$

for $u = 1, \ldots, U, \ n = 1, \ldots, N$

$$\begin{bmatrix} c_v[1] \\ c_v[2] \\ \vdots \\ c_v[\mathrm{N}] \end{bmatrix} = \begin{bmatrix} \omega_v[1] \\ \omega_v[2] \\ \vdots \\ \omega_v[\mathrm{N}] \end{bmatrix} \cdot \theta_v + \begin{bmatrix} \psi_v[1] \\ \psi_v[2] \\ \vdots \\ \psi_v[N] \end{bmatrix} \tag{S6}$$

$$\text{for } v = 1,\ldots,V,\ n = 1,\ldots,N$$

The equations (S5) and (S6) are simply represented as the following formulas:

$$L_u = \Gamma_u \cdot \theta_u + \Xi_u,\ \text{for } u = 1,\ldots,U \tag{S7}$$

$$C_v = \Gamma_v \cdot \theta_v + \Xi_v,\ \text{for } v = 1,\ldots,V \tag{S8}$$

The parameter vectors θ_u and θ_v are estimated by solving the constrained linear least-squares parameter estimation problem via the MATLAB optimization toolbox as follows:

$$\hat{\theta}_u = \arg\min_{\theta_u} \frac{1}{2} \left\| \Gamma_u \cdot \theta_u - L_u \right\|_2^2 \tag{S9}$$

$$\text{subject to} \underbrace{\begin{bmatrix} 0 & \cdots & \cdots & 0 \\ \vdots & \ddots & & \vdots \\ \vdots & & \ddots & \vdots \\ 0 & \cdots & \cdots & 0 \end{bmatrix}}_{\mathrm{F}_u} \left| \underbrace{\begin{matrix} 0 & \cdots & \cdots & 0 \\ \vdots & \ddots & & \vdots \\ \vdots & & \ddots & \vdots \\ 0 & \cdots & \cdots & 0 \end{matrix}}_{\mathrm{G}_u} \right| \underbrace{\begin{matrix} 1 & 0 & \cdots & 0 \\ 0 & \ddots & \ddots & \vdots \\ \vdots & \ddots & \ddots & 0 \\ 0 & \cdots & 0 & 1 \end{matrix}}_{\mathrm{H}_u} \left| \begin{matrix} 0 \\ \vdots \\ \vdots \\ 0 \end{matrix} \right] \theta_u \le \begin{bmatrix} 0 \\ \vdots \\ \vdots \\ 0 \end{bmatrix}$$

$$\hat{\theta}_v = \arg\min_{\theta_v} \frac{1}{2} \left\| \Gamma_v \cdot \theta_v - C_v \right\|_2^2 \tag{S10}$$

$$\text{subject to} \underbrace{\begin{bmatrix} 0 & \cdots & \cdots & 0 \\ \vdots & \ddots & & \vdots \\ \vdots & & \ddots & \vdots \\ 0 & \cdots & \cdots & 0 \end{bmatrix}}_{\mathrm{F}_v} \left| \underbrace{\begin{matrix} 0 & \cdots & \cdots & 0 \\ \vdots & \ddots & & \vdots \\ \vdots & & \ddots & \vdots \\ 0 & \cdots & \cdots & 0 \end{matrix}}_{\mathrm{G}_v} \right| \underbrace{\begin{matrix} 1 & 0 & \cdots & 0 \\ 0 & \ddots & \ddots & \vdots \\ \vdots & \ddots & \ddots & 0 \\ 0 & \cdots & 0 & 1 \end{matrix}}_{\mathrm{H}_v} \left| \begin{matrix} 0 \\ \vdots \\ \vdots \\ 0 \end{matrix} \right] \theta_v \le \begin{bmatrix} 0 \\ \vdots \\ \vdots \\ 0 \end{bmatrix}$$

The constraint conditions on the least-squares parameter estimation problem in (S9) and (S10) mean the estimated post-transcriptional regulatory abilities of miRNAs on lncRNAs and miRNAs are guaranteed to be negative.

The parameters of the candidate GWGEN of MIBC and ABC were estimated from the corresponding microarray data. Because the different experimental conditions may cause errors in data from various databases, we utilized Akaike Information Criterion (AIC) to prune the false positives and detected the system order (the number of interactions or regulations) of the real GWGENs. The equations of AIC for the u-th lncRNA and the v-th miRNA can be described as follows:

$$AIC(F_u, G_u, \mathrm{H}_u) = \log(\hat{\Phi}_u^2) + \frac{2(\Omega_u + 1)}{N},$$
$$\text{where } \hat{\Phi}_u = \sqrt{\frac{(G_u - (\Gamma_u \cdot \hat{\theta}_u))^T (G_u - (\Gamma_u \cdot \hat{\theta}_u))}{N}} \quad , \Omega_u = F_u + G_u + \mathrm{H}_u \tag{S11}$$

where $\hat{\Phi}_u$ and Ω_u individually denote the estimated residual error and the number (system order) of regulations for the parameter estimation problem (S9) in the u-th lncRNA; $\hat{\theta}_u$ represents the estimated parameter vector for the u-th lncRNA by (S9).

$$AIC(F_v, G_v, \mathrm{H}_v) = \log(\hat{\Phi}_v^2) + \frac{2(\Omega_v + 1)}{N},$$
$$\text{where } \hat{\Phi}_v = \sqrt{\frac{(G_v - (\Gamma_v \cdot \hat{\theta}_v))^T (G_v - (\Gamma_v \cdot \hat{\theta}_v))}{N}} \quad , \Omega_v = F_v + G_v + \mathrm{H}_v \tag{S12}$$

where $\hat{\Phi}_v$ and Ω_s individually indicate the estimated residual error and the number (system order) of regulations for the parameter estimation problem (S10) in the v-th miRNA; $\hat{\theta}_v$ represents the estimated parameter vector for the t-th miRNA by (S10).

To obtain the real system order in the candidate GWGEN, the AIC can be minimized by the following system order detection method:

$$F_u^*, G_u^*, H_u^* = \underset{F_u, G_u, H_u}{\operatorname{argmin}} AIC(F_u, G_u, H_u), \text{ for } u = 1, \ldots, U \tag{S13}$$

$$F_v^*, G_v^*, H_v^* = \underset{F_v, G_v, H_v}{\operatorname{argmin}} AIC(F_v, G_v, H_v), \text{ for } v = 1, \ldots, V \tag{S14}$$

Where F_u^*, G_u^*, H_u^* denote the real number of regulations by TFs, lncRNAs and miRNAs on the u-th lncRNA, respectively; F_v^*, G_v^*, H_v^* denote the real number of regulations by TFs, lncRNAs and miRNAs on the v-th miRNA, respectively.

Consequently, the regulations out of the real system order by the AIC in (S13) and (S14) are considered false positives which would be pruned away to obtain the real GWGENs of MIBC and ABC.

S.2 Tables

Table S1. The statistics of nodes in the candidate GWGEN and the real GWGENs of MIBC and ABC after system identification by their genome-wide microarray data

Nodes	Candidate GWGEN	Real GWGEN (MIBC)	Real GWGEN (ABC)
Proteins	14,339	11,755	11,749
Receptors	2452	2358	2356
TFs	1611	1569	1571
miRNAs	217	113	119
LncRNAs	5487	2782	2783
Total node	24,106	18,577	18,578

NOTE: Nodes are defined as the proteins/genes that have at least one interaction or regulation with others in the GWGEN.

Table S2. The statistics of edges in the candidate GWGEN and the real GWGENs of MIBC and ABC after system identification by their genome-wide microarray data

Edges	Candidate GWGEN	Real GWGEN (MIBC)	Real GWGEN (ABC)
PPI edge	6,121,221	1,904,121	1,570,962
TF-protein	95,157	5294	5379
TF- receptor	10,341	1102	1079
TF-TF	38,182	2459	2914
TF- miRNA	73	6	11
TF- LncRNA	4678	1588	1661
miRNA-protein	1375	488	514
miRNA-miRNA	24	2	5
miRNA-LncRNA	3605	294	312
LncRNA-protein	20,935	7922	8298
LncRNA- receptor	131	51	41
LncRNA-LncRNA	12,163	5649	5897
Total edge	6,307,885	1,928,976	1,597,073

NOTE: Edges are defined as interactions or regulations between two nodes and are denoted by "node1-node2".

Table S3. The KEGG pathway enrichment analysis of core GWGEN of MIBC using the DAVID tool

Pathway Analysis	Gene Numbers	*p*-value
Wnt signaling pathway	69	6.8E-4
PI3K-Akt signaling pathway	131	7.8E-4
NF-kappa B signaling pathway	43	7.9E-3
MAPK signaling pathway	102	2.4E-2

Table S4. The KEGG pathway enrichment analysis of core GWGEN of ABC using the DAVID tool

Pathway Analysis	Gene Numbers	*p*-value
Wnt signaling pathway	70	2.4E-4
Pathways in cancer	183	1.8E-3
MAPK signaling pathway	104	8.3E-3
Notch signaling pathway	26	1.6E-2
FoxO signaling pathway	47	6.0E-2

Table S5. Model performance of the DNN-based DTI model with 5-fold cross-validation (epoch=54)

	Validation loss	Validation accuracy	Testing loss	Testing accuracy
1	0.186351	0.930611	0.214089	0.925667
2	0.211873	0.930700	0.207743	0.933243
3	0.229786	0.933277	0.209450	0.930524
4	0.222162	0.929363	0.198727	0.931541
5	0.195523	0.932565	0.192000	0.932143
Average	0.209139	0.931303	0.204402	0.930624
Standard Deviation	0.016174	0.001421	0.007956	0.002630

S.3 Figures

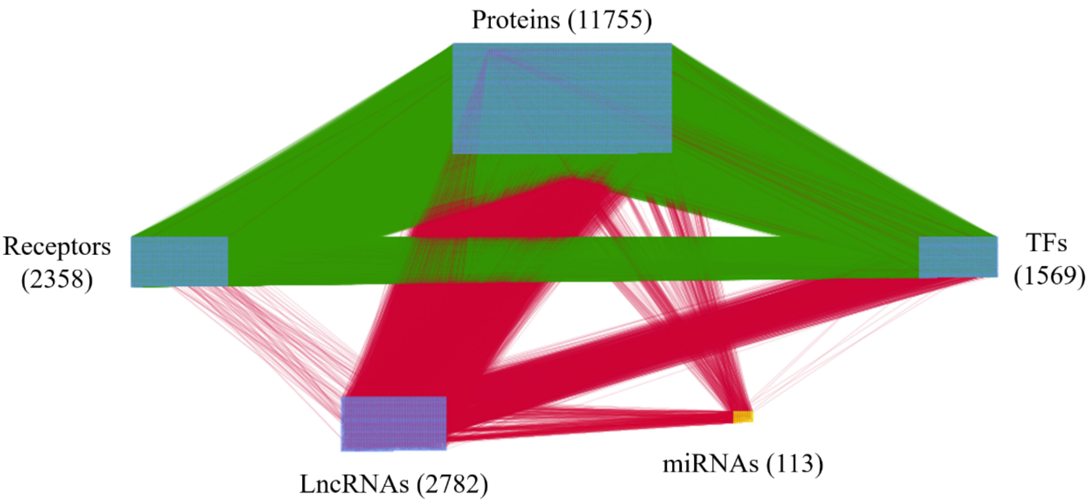

Figure S1. The real GWGEN of MIBC. The green lines represent the protein-protein interactions and the red lines indicate the gene regulations. The node numbers of proteins, receptors, TFs, LncRNAs and miRNAs are 11755, 2358, 1569, 2782 and 113, respectively.

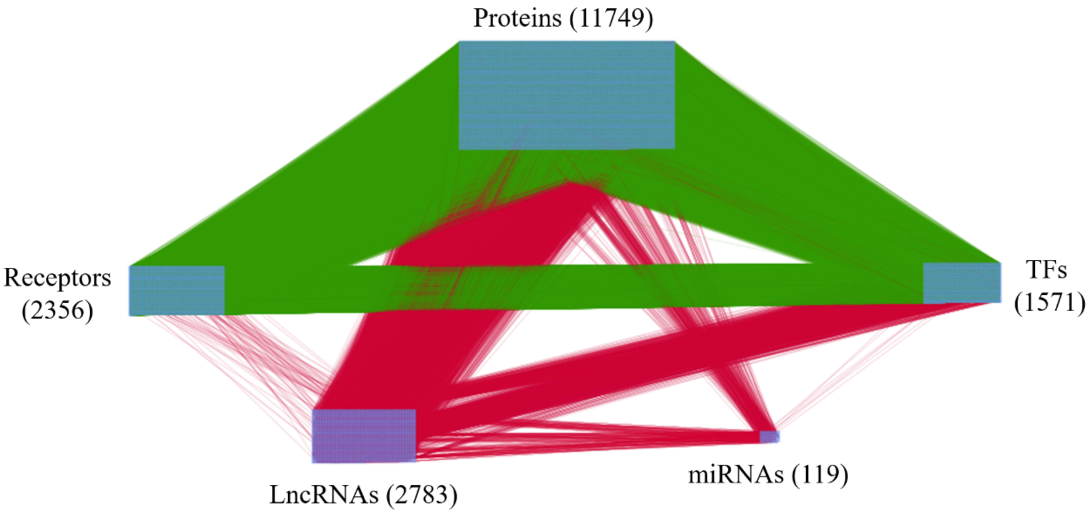

Figure S2. The real GWGEN of ABC. The green lines represent the protein-protein interactions and the red lines indicate the gene regulations. The node numbers of proteins, receptors, TFs, LncRNAs and miRNAs are 11749, 2356, 1571, 2783 and 119, respectively.

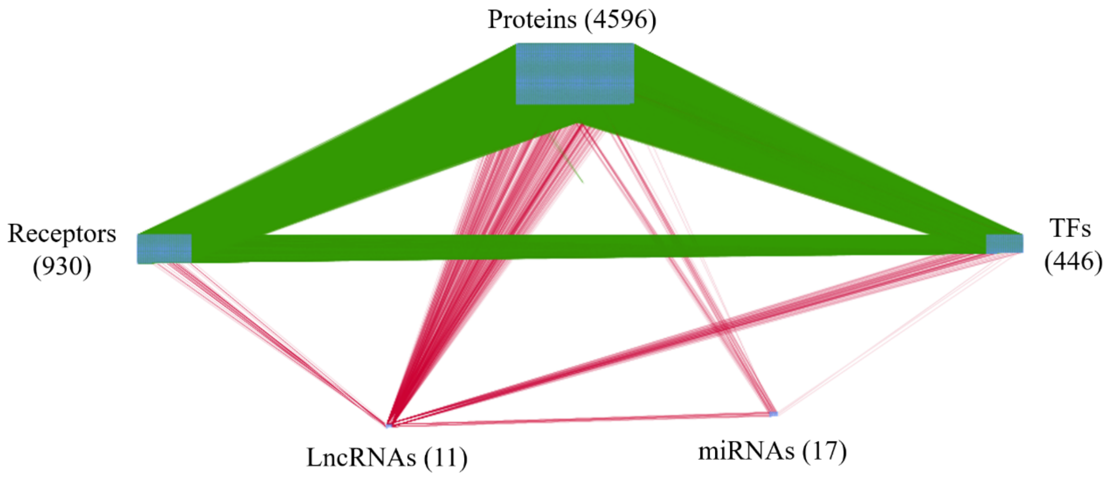

Figure S3. The core GWGEN of MIBC. The green lines represent the protein-protein interactions and the red lines indicate the gene regulations. The node numbers of proteins, receptors, TFs, LncRNAs and miRNAs are 4596, 930, 446, 11 and 17, respectively.

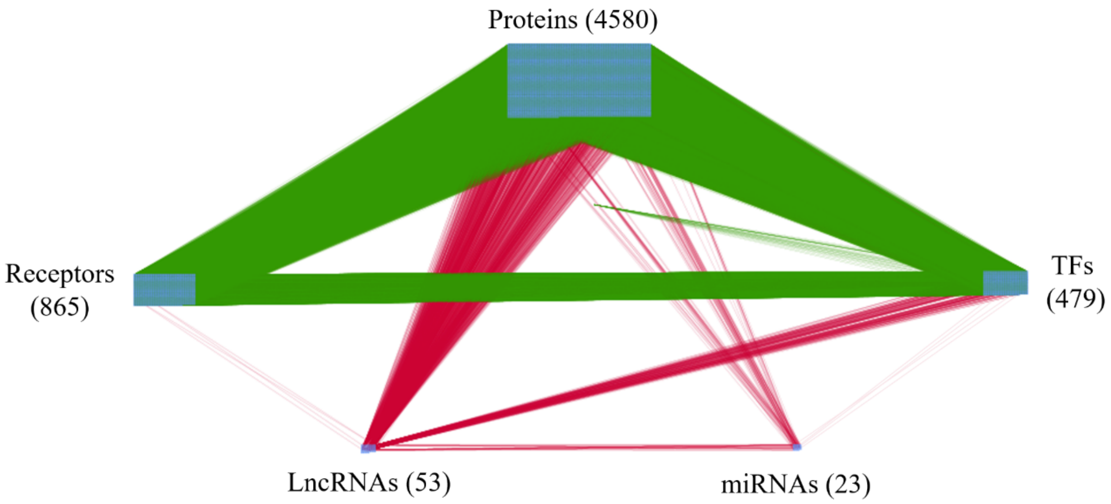

Figure S4. The core GWGEN of ABC. The green lines represent the protein-protein interactions and the red lines indicate the gene regulations. The node numbers of proteins, receptors, TFs, LncRNAs and miRNAs are 4580, 865, 479, 53 and 23, respectively.

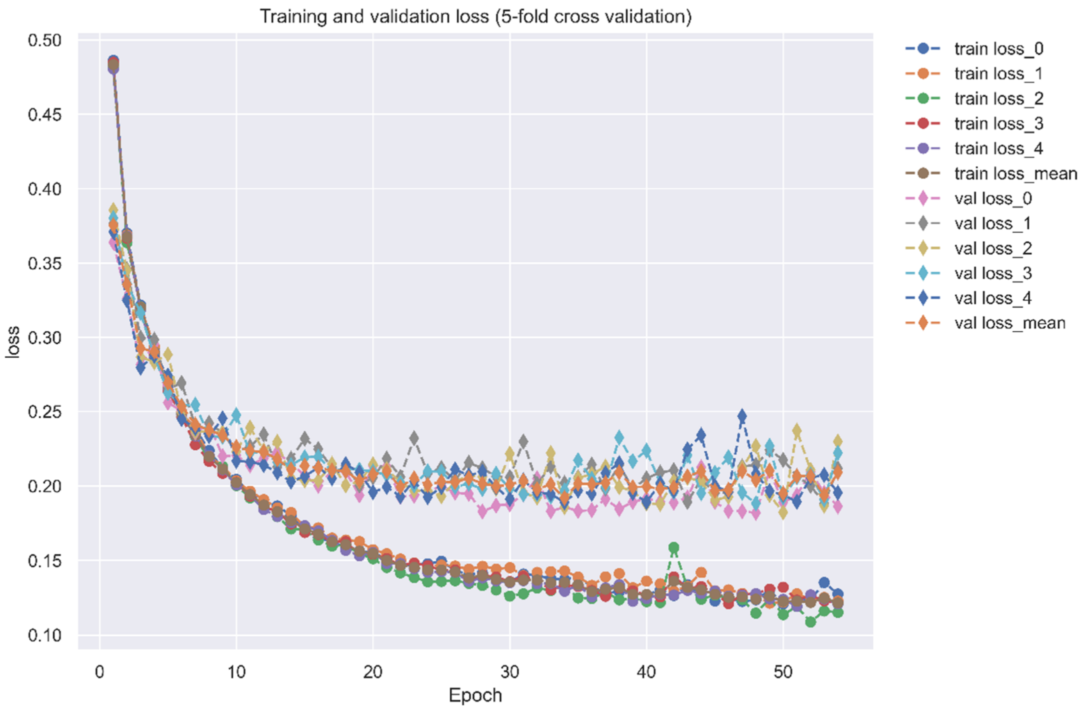

Figure S5. Training and validation loss of DNN-based DTI model (5-fold cross validation). The early stopping strategy is used to automatically stop the learning process (at epoch = 54).

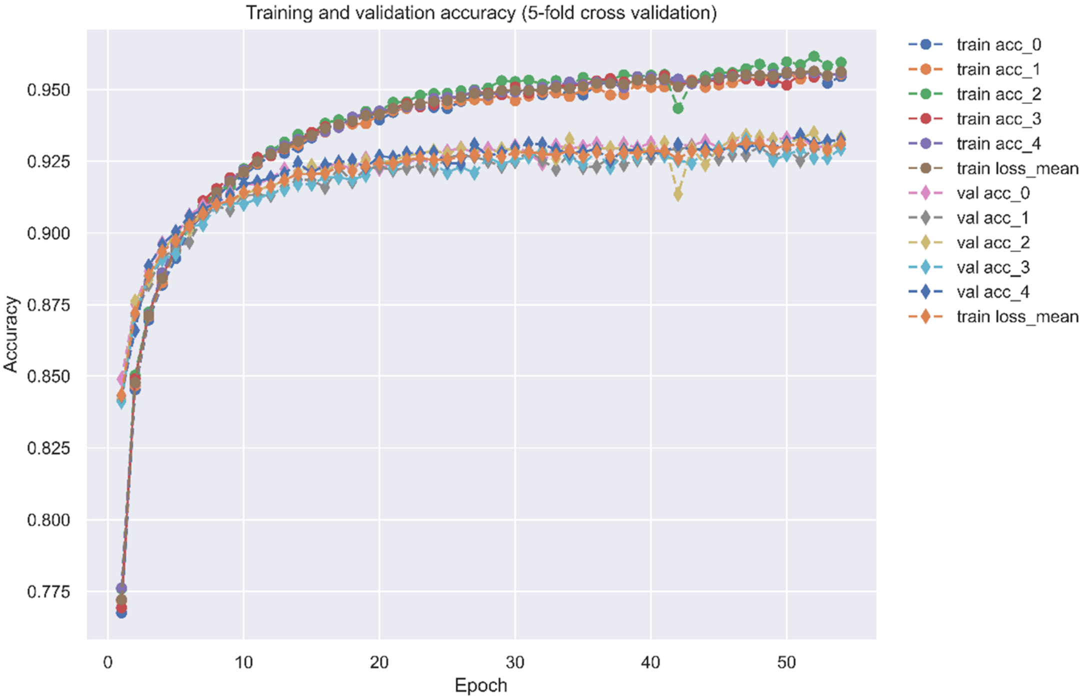

Figure S6. Training and validation accuracy of DNN-based DTI model (5-fold cross-validation). The early stopping strategy is used to automatically stop the learning process (at epoch = 54).

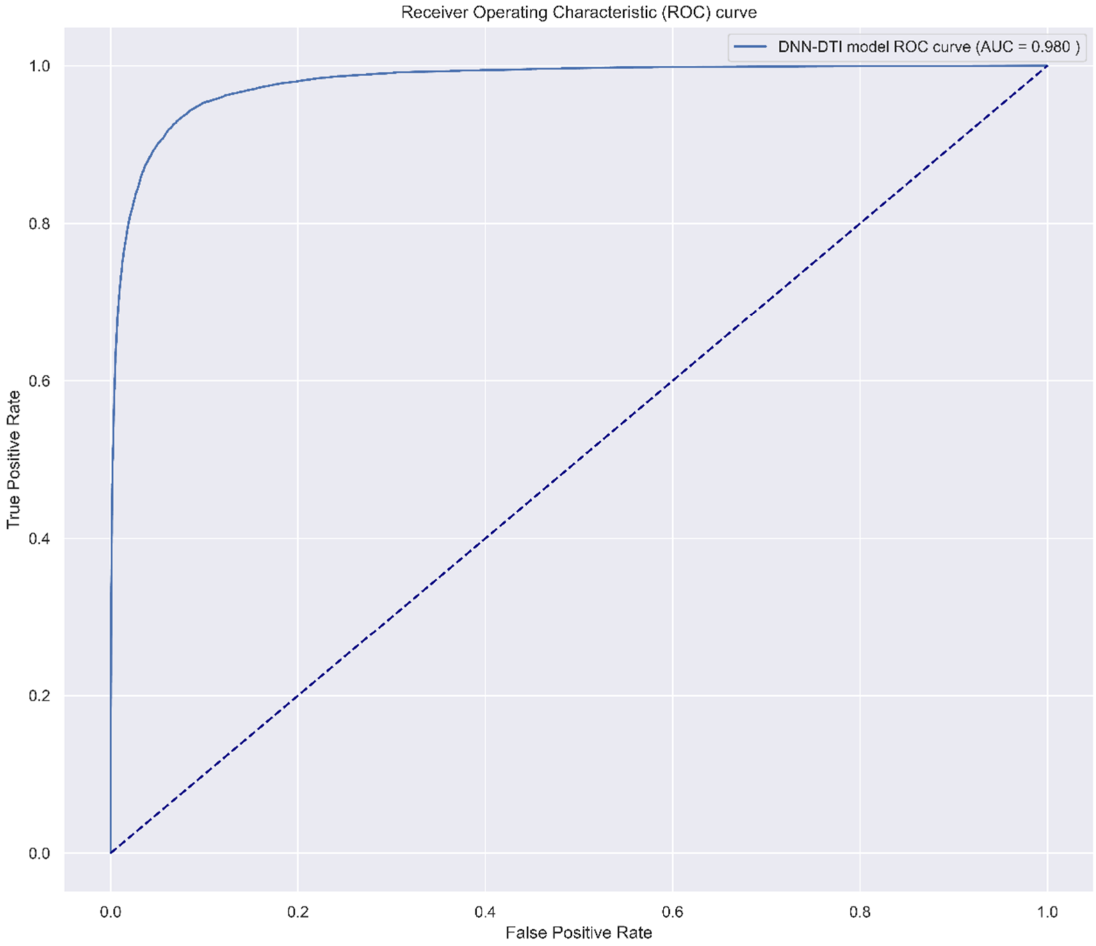

Figure S7. The Receiver Operating Characteristic (ROC) curve and the Area Under the Curve (AUC) score of the DNN-based DTI model. The dashed line on the diagonal denotes that the model is a random prediction, which can be used to judge the quality of the model. On the upper left of the dashed line, the DNN-based DTI model (AUC = 0.980) outperforms the random prediction model (AUC = 0.5).
